# Propagation of Orientation Across Lengthscales in Sheared Self‐Assembling Hierarchical Suspensions via Rheo‐PLI‐SAXS

**DOI:** 10.1002/advs.202410920

**Published:** 2024-12-25

**Authors:** Reza Ghanbari, Ann Terry, Sylwia Wojno, Marko Bek, Kesavan Sekar, Amit Kumar Sonker, Kim Nygård, Viney Ghai, Simona Bianco, Marianne Liebi, Aleksandar Matic, Gunnar Westman, Tiina Nypelö, Roland Kádár

**Affiliations:** ^1^ Department of Industrial and Materials Science, Division of Engineering Materials Chalmers University of Technology Gothenburg SE‐412 96 Sweden; ^2^ MAX IV Laboratory Lund University Lund SE‐224 84 Sweden; ^3^ LINXS Institute of advanced Neutron and X‐ray Science (LINXS) Scheelevägen 19 Lund 223 70 Sweden; ^4^ Wallenberg Wood Science Centre (WWSC) Chalmers University of Technology Gothenburg SE‐412 96 Sweden; ^5^ School of Chemistry University of Glasgow Glasgow G12 8QQ UK; ^6^ Department of Physics, Division of Materials Physics Chalmers University of Technology Gothenburg SE‐412 96 Sweden; ^7^ Paul Scherrer Institute Division of Photon Science Villigen CH‐5232 Switzerland; ^8^ Institute of Materials Ecole Polytechnique Fédérale de Lausanne (EPFL) Lausanne CH‐1015 Switzerland; ^9^ Department of Chemistry and Chemical Engineering, Division of Organic Chemistry Chalmers University of Technology Gothenburg SE‐412 96 Sweden; ^10^ Department of Chemistry and Chemical Engineering, Division of Applied Chemistry Chalmers University of Technology Gothenburg SE‐412 96 Sweden; ^11^ Department of Bioproducts and Biosystems Aalto University Espoo FI‐00760 Finland; ^12^ Present address: NKT Technology Consulting VästerÄs Sweden; ^13^ Present address: VTT Technical research center of Finland Biomaterial processing and products Tietotie 4E Espoo 02150 Finland

**Keywords:** advanced rheological techniques, cellulose nanocrystals, liquid crystalline suspensions, multiscale orientation, polarized light imaging, small‐angle X‐ray scattering

## Abstract

Simultaneous rheological, polarized light imaging, and small‐angle X‐ray scattering experiments (Rheo‐PLI‐SAXS) are developed, thereby providing unprecedented level of insight into the multiscale orientation of hierarchical systems in simple shear. Notably, it is observed that mesoscale alignment in the flow direction does not develop simultaneously across nano‐micro lengthscales in sheared suspensions of rod‐like chiral‐nematic (meso) phase forming cellulose nanocrystals. Rather, with increasing shear rate, orientation is observed first at mesoscale and then extends to the nanoscale, with influencing factors being the aggregation state of the hierarchy and concentration. In biphasic systems, where an isotropic phase co‐exists with self‐assembled liquid crystalline mesophase domains, the onset of mesodomain alignment towards the flow direction can occur at shear rates nearing one decade before a progressive increase in preferential orientation at nanoscale is detected. If physical confinement prevents the full formation of a cholesteric phase, mesoscale orientation occurs in shear rate ranges that correspond to de‐structuring at nanoscale. Interestingly, nano‐ and mesoscale orientations appear to converge only for biphasic suspensions with primary nanoparticles predominantly made up of individual crystallites and in a high‐aspect ratio nematic‐forming thin‐wall nanotube system. The nano‐micro orientation propagation is attributed to differences in the elongation and breakage of mesophase domains.

## Graphical abstract

A simultaneous rheological, polarized light imaging, and small‐angle X‐ray scattering experiments (Rheo‐ PLI‐SAXS) is presented. Thereby, unprecedented level of insight into the multiscale orientation of hierarchical systems in simple shear is reached. Notably, mesoscale alignment in the flow direction does not develop simultaneously across nano‐micro lengthscales in sheared suspensions of rod‐like chiral‐nematic (meso) phase forming cellulose nanocrystals.

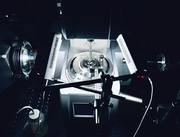

## 1 Introduction

Many functional materials are hierarchical, exhibiting properties that are governed by the structure at several different length scales. A common major challenge in forming such hierarchies into materials of suitable properties is controlling the arrangement of (supramolecular) hierarchy levels through techniques that are suitable for high throughput.^[^ ^1^, ^2^ ^]^ While self‐assembly of the primary particles alone could be one route for certain applications, whenever possible, material structural control using flow based methods is second to none in terms of throughput. Recently, flow‐based techniques have been used to create high performance fibers from cellulose nanofibrils,^[^ ^3^ ^]^ photonic films from cellulose nanocrystals^[^ ^4^ ^]^ etc. However, the multiscale orientation and structuring dynamics in such systems in flow has not been previously investigated simultaneously across nano‐micro lengthscales. This is perhaps a most critical step in understanding how to control structure development through flow, similar to how hierarchical control in synthetic polymers has been achieved in the past decades by tuning molecular properties and their interaction with flow and thermal fields during forming operations.^[^ ^5^ ^]^

**Figure 1:**
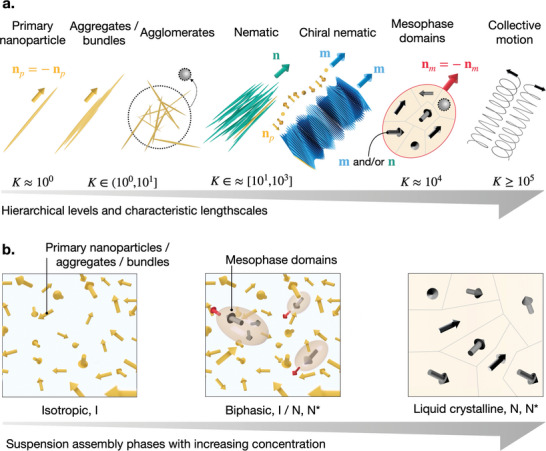
a) Typical hierarchical levels in self‐assembling systems composed of rod‐like nanoparticles in flow. The collective (ordered) motion refers to complex director fields, i.e., flow instabilities, where hierarchical components move along the trajectory of seconday flows. *K* is the ratio between the characteristic lengthscale the level of the hierarchy and a characteristic lengthscale of the primary particle. b) Illustration of assembly phases with increasing primary nanoparticle concentration. Within the mesoscale domains, directors are represented in grayscale as a generic representation of mesocrystals, nematic (N) or chiral nematic (N*). Nanoparticle **n**_*p*_, nematic **n** and mesophase **n**_*m*_ directors are pseudovectors (e.g., **n** = −**n**).

Taking as example rod‐like nanoparticles and limiting the discussion to levels of the hierarchy relevant to the experiments presented, hierarchy in such nanostructured self‐assembling systems can comprise,^[^ ^6^, ^7^ ^]^ **Figure** **1**a: the primary nanoparticles (cellulose nanocrystals; CNCs), aggregates and bundles thereof, agglomerates, nematic and chiral nematic structures, and domains (mesophase) thereof. Furthermore, the onset of flow instabilities/secondary flows leads to complex director fields, where certain structural elements have a collective complex motion^[^ ^8^ ^]^ The different dimensions of the hierarchy are represented in the figure relative to the dimensions of the primary particles ($K=L/L_{ref}$, where L is a characteristic lengthscale of a level of the hierarchy and $L_{ref}$ a characteristic lengthscale of the primary nanoparticle). Three assembly phases are generally distinguished for materials with such hierarchies, Figure 1b: an isotropic phase (I) at low concentrations comprising individual nanorods and their bundles/agglomerates; a biphasic phase (I/N,N*) at concentrations above a critical self‐assembly concentration^[^ ^9^ ^]^ where the isotropic phase coexists with mesophase (liquid crystalline) domains formed by one (tactoids) or more nematic (N) and/or chiral nematic (N*) structures; and a liquid crystalline phase (N,N*) at concentrations sufficiently high such that the isotropic phase is absent. Importantly, when referring to the material nanoscale structure we refer to the individual nanorods in the isotropic phase or those contained within the liquid‐crystalline domains, in the case of biphasic and liquid crystalline phase.

From a materials characterization point of view, resolving the flow‐induced hierarchical structures in Figure 1 remains a challenge. For example, rheology is a powerful analytical tool to probe material structure using viscometric flows, while also being of high relevance for the design and understanding of fabrication methods.^[^ ^10^, ^11^ ^]^ However, rheological material functions, such as the shear viscosity, are a bulk or average measure of all levels of the hierarchy. Therefore, a separation of the individual contributions of the hierarchy is not directly possible, especially in nonlinear viscoelastic conditions. By nonlinear conditions, we refer here to the shear rate dependent regions of viscosity functions. Therefore, various hyphenated (combined) techniques have been developed to try and correlate bulk behavior to material lengthscales, especially at nanoscale. Taking as examples a few rheometer‐based hyphenated techniques applied to CNC suspensions,^[^ ^12^ ^]^ state‐of‐the‐art combined rheological and small‐angle scattering experiments have previously been complemented by separate birefringence experiments on shear cells^[^ ^13^ ^]^ and light scattering experiments in flow channels^[^ ^14^ ^]^ in order to interpret the results. Thus, regardless of whether optical, spectroscopic, scattering, rheological or combinations thereof is used, analytical techniques usually require cross‐checking different methods in order to understand the hierarchy. A more extensive discussion of characteristic material or characterization lengthscales can be found in Section S3 (Supporting Information).

Since Maxwell's 1873 first landmark observations,^[^ ^15^ ^]^ polarized light imaging (PLI) of flows, or shear‐induced polarized light imaging, has remained a simple yet powerful method for interrogating the microstructure of materials in flow.^[^ ^12^, ^16^, ^17^, ^18^ ^]^ Light–matter interactions in a material that cause an anisotropic propagation of light, such as the liquid crystalline assemblies in Figure 1a, can be visualized using two linear polarizers in the form of interference colors. Despite its ease‐of‐use, the interpretation of PLI visualizations is not trivial and can contain contributions from both nano and mesoscale levels of the hierarchy.^[^ ^18^, ^19^, ^20^, ^21^, ^22^, ^23^ ^]^ Considering a simple shear flow in a parallel‐plate measuring geometry, PLI visualizations in the plane of shear can measure orientation^[^ ^12^, ^18^ ^]^ (via the onset of Maltese‐cross patterns) and provide evidence of secondary flows^[^ ^8^, ^12^, ^18^, ^22^, ^23^ ^]^ (via flow‐induced birefringence textures). Although not directly emphasized, it is often inferred that shear‐induced orientation as observed through Rheo‐PLI is representative of nanoscale orientation,^[^ ^20^, ^22^, ^24^ ^]^ or, conversely, orientation of the primary particles is considered to be representative of the orientation and elongation of the liquid‐crystalline mesophase.^[^ ^13^, ^14^ ^]^ Small‐angle X‐ray scattering (SAXS), in turn, probes lengthscales that span a few nanometers to a few hundreds of nanometers, depending on the choice of scattering vector, **q**, range.^[^ ^25^ ^]^ While combinations of rheology and PLI (Rheo‐PLI) or SAXS (Rheo‐SAXS) are relatively accessible, a Rheo‐PLI‐SAXS experiment has not been previously performed, to the best of our knowledge. Without simultaneously considering PLI and SAXS, the exact lengthscales that contribute to PLI Maltese‐cross and textures could not be interrogated. With the advent of fourth‐generation synchrotrons and the ensuing 100‐fold increase in brightness of the X‐ray beam,^[^ ^26^ ^]^ we are in the position to overcomes issues related to signal attenuation in a parallel‐plate measuring geometry. Here, we report on the development of simultaneous, multiscale Rheo‐PLI‐SAXS and its application to flow‐induced mesophase nano‐micro orientation in hierarchical systems. We focus on cholesteric phase‐forming cellulose nanocrystal suspensions, surface modified CNCs, and nematic‐phase forming thin‐wall nanotubes, observing an intriguing array of previously inaccessible phenomena.

## 2 Results and Discussion

### 2.1 Multiscale Orientation Analysis from Rheo‐PLI‐SAXS

The Rheo‐PLI‐SAXS experiments discussed below are based on a custom design parallel‐plate measuring system, see Figure 5a. Additionally, complementary Rheo‐SAXS measurements were performed on concentric cylinder and cone‐plate measuring geometries, see Figure S1 and Section S3.1 (Supporting Information).

**Figure 2:**
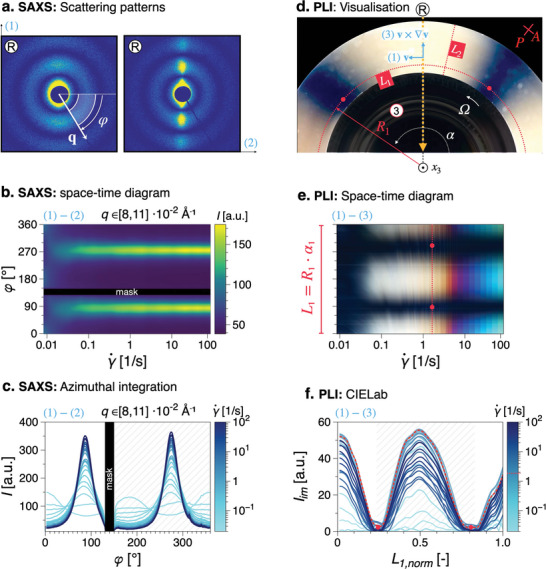
Overview of the Rheo‐PLI‐SAXS experiment: a) Examples of nearly‐isotropic and strongly anisotropic SAXS scattering patterns; b) azimuthal integration of SAXS data in the form of space‐time diagrams with shear rate as the independent variable; c) azimuthal integration data in the form of scattering intensity, *I* as function of the azimuthal angle, highlighting scattering peaks used for orientation analysis (hatched) d) example of PLI e) space‐time diagrams formed by extracting and concatenating pixel lines *L*_1_ and *L*_2_ from each frame of PLI video recordings. *L*_1_ corresponds to the geometrical locus where the local shear rate corresponds to the nominal shear rate of the rheometric experiment. f) PLI the norm in the CIELab color space norm, *I*_*im*_, defined in Equation (1) in the Supporting Information, as function of the normalized *L*_1_, with a similar hatched highlight of a peak region used for analysis. Coordinates (1), (2), (3) correspond to the velocity vector, **v**, velocity gradient, ∇v and vorticity, ∇×v, directions, respectively. (R) refers to the radial incident X‐ray configuration. Note: the data in a–c and d–f do not correspond to each other.

SAXS patterns, **Figure** **2**a, can be azimuthally integrated in the form of space‐time scalar plots, Figure 2b, for a visual evaluation of the scattering anisotropy during flow. By fitting the scattering intensity (a.u.), over the hatched region in Figure 2c, with a Legendre polynomial series expansion, the Hermans orientation parameter,^[^ ^27^ ^]^ 〈*P*_2_〉, can be determined, Equation (2).^[^ ^13^, ^28^, ^29^ ^]^ For integration limits set to [0, π] in Equation (2), 〈*P*_2_〉_*SAXS*_ ∈ [− 0.5, 1], with 〈*P*_2_〉_*SAXS*_〉0 signifying preferential orientation perpendicular to the flow direction, 〈*P*_2_〉_*SAXS*_ = 0 random orientation and 〈*P*_2_〉_*SAXS*_ < 0 preferential orientation in the flow direction. The limiting cases assume a perfect orientation in the respective directions, e.g., –0.5 represents a full orientation at nanoscale in the flow direction. This interpretation refers to an average of the 3D volume of fluid interrogated by the incident beam. The preferred orientation angle corresponding to the nanoscale anisotropy can be then determined from the peak maxima in the reciprocal space.

As already mentioned, qualitatively, orientation in the flow direction from cross‐polarized PLI can be observed by the onset of the so‐called Maltese‐cross pattern, see the example in Figure 2d. Thereby, optical indicatrixes uniformly oriented in the flow direction cause extinction regions in a cross polarized PLI setup whenever one of the refractive indices of the indicatrix is parallel to the polarization direction of the polarizers.^[^ ^18^, ^19^ ^]^ We note that the notion of optical indicatrix is not intrinsically associated with any particular level of the hierarchy. Taking an arc of length *L*_1_ at a radius *R*_1_ = 2*R*/3, where *R* is the radius of the measuring plate (2 · *R* = 43 mm), the onset of the Maltese‐cross pattern can be readily observed in the form of (*L*_1_, *t*) space‐time diagrams, Figure 2e. The arc *L*_1_ corresponds to the geometrical loci in the flow domain where the local shear rate equals the nominal shear rate, $\dot{\gamma }\left(r\right)=\dot{\gamma }$, see Section S3.1 (Supporting Information). To quantify the imaging data, RGB color intensities were converted to the CIELab colorspace,^[^ ^30^ ^]^ which is designed to match human visual perception, see Figure S2 for an illustration of the colorspace. The norm of the position vector in the color space, *I*_*im*_, see Equation (1), was used for quantitative analysis and is plotted in Figure 2f. It can be seen that the data is qualitatively similar to the SAXS azimuthal integration data in Figure 2c. Therefore, the same data analysis framework can be used to determine the onset of a peak in the PLI data, with the important distinction that the physical interpretation is not equivalent. For integration limits set to [− π/2, π/2] in Equation (2), and replacing the scattering intensity *I* with *I*_*im*_, we define *P*_2 *PLI*_ as a measure of the onset of the Maltese‐cross pattern: *P*_2 *PLI*_ > 0 signifies the onset of the Maltese‐cross pattern, i.e., preferential orientation in the flow direction. To asses whether a Maltese‐cross pattern may occur at $\dot{\gamma }\left(r>2R/3\right)$, analogous (*L*_2_, *t*) (refer to Figure 2d) diagrams are also plotted.

In the following, we show that the onset of preferential orientation in the flow direction as observed by PLI and SAXS can show a significant discrepancy in critical shear rate. We explore this discrepancy for suspensions as function of their primary particle aggregation state, including through surface modification, assembly phase and whether the systems form chiral nematic or nematic mesophase. We interpret this orientation discrepancy as indication that PLI captures the orientation of mesoscale domains and that nanoparticles within these mesodomains follow mesoscale orientation only in certain conditions which are likely related to the elongation of mesodomains in the flow direction.

### 2.2 Biphasic CNC Suspensions

**Figure 3:**
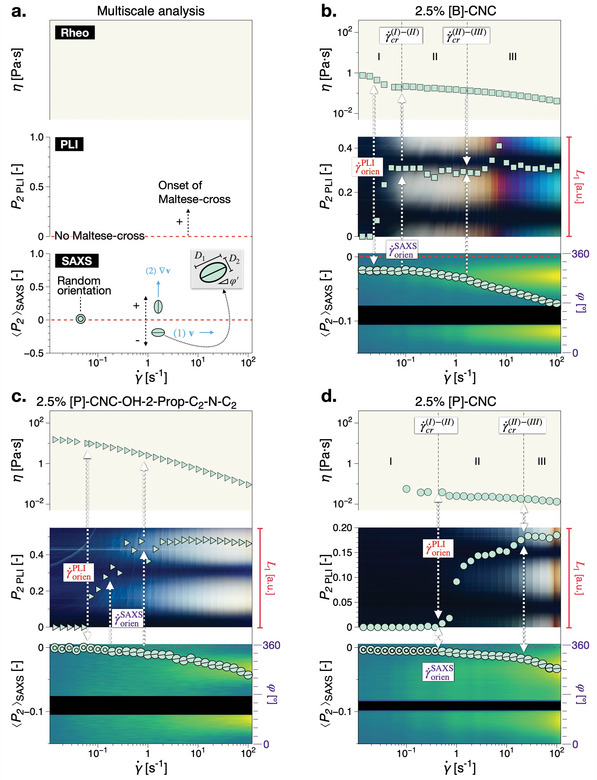
Multiscale analysis of biphasic CNC suspensions: a) Overview of comparative data analysis from Rheo‐PLI‐SAXS: (bottom) overlay of scattering intensity (arbitrary units) from azimuthal integration as function of time/shear rate, $\dot{\gamma }$, and the corresponding Hermans orientation parameter, ${\langle P_{2}\rangle }_{SAXS}\left(\dot{\gamma }\right)$; (middle) overlay of PLI space–time diagram, *L*_1_ vs. $\dot{\gamma }$ and the onset of the Maltese‐cross pattern as quantified by Hermans' algorithm to the PLI data, $P_{2\;PLI}\left(\dot{\gamma }\right)$; (top) steady shear viscosity functions, $\eta (\dot{\gamma })$. *D*_1, 2_ scale with |〈*P*_2_〉|, see Equation (S11, Supporting Information). Multiscale analysis for b) 2.5% [B]‐CNC, c) 2.5% [P]‐CNC‐OH‐2‐Prop‐C_2_‐N‐C_2_, and d) 2.5% [P]‐CNC.

We consider first biphasic systems, i.e., where self‐assembled liquid‐crystalline domains co‐exist with an isotropic phase, see Figure 1b. Three CNC suspensions of equal concentration, 2.5% CNC, but which differ in the aggregation state of the primary nanoparticles, are compared in **Figure** **3**. A general explanation of the diagrams used for multiscale analysis can be found in Figure 3a. Prefix [B] indicates that the CNCs are found predominantly in bundles and aggregates thereof and [P] that CNC crystallites are prevalent in the suspensions. This is confirmed by the transmission electron (TEM) and polarized optical microscopy (POM) analysis in Figure S7 (Supporting Information). The difference between the two types CNC suspensions stems from the preparation procedure, with CNC crystallites accessible through probe sonication.^[^ ^2^ ^]^ We had previously estimated the aspect ratios of [B]‐CNCs to be between 25 and 75,^[^ ^31^ ^]^ while sonication doses comparable to those applied for preparing [P] (ca. 15 kJ mL^−1^) have been reported to reduce the (mean effective 3D) aspect ratio by approximately 25%.^[^ ^7^ ^]^

The suspension in Figure 3b exhibits a so‐called three‐region behavior in the viscosity function and order parameter. The “three regions” refer to three distinct slopes in steady shear viscosity functions. Albeit rather ubiquitous in liquid crystalline systems, the fundamental understanding and control thereof remains a challenge.^[^ ^12^ ^]^ In the present data analysis, whenever present, the three regions were highlighted based on the Hermans orientation parameter from SAXS, 〈*P*_2_〉_SAXS_, as it has been shown to be a more sensitive measure thereof in comparison to the viscosity functions.^[^ ^13^ ^]^ The three regions have been broadly attributed to (I) slow dynamics of the mesophase, (II) a progressive breakup of the cholesteric phase into nematic domains and (iii) eventually developing into a paranematic monodomain phase accompanied by strong orientation in the flow direction.^[^ ^13^ ^]^ We note that in the most recent investigations on the topic, Pignon and co‐workers^[^ ^14^ ^]^ have assigned the progressive breakup of large liquid crystalline domains into sub‐micrometer sized tactoids to region (I). In the multiscale analysis diagrams, the critical shear rates corresponding to the transitions between the regions are here labeled as ${\dot{\gamma }}_{cr}^{\left(I)-(II\right)}$ and ${\dot{\gamma }}_{cr}^{\left(II)-(III\right)}$, respectively. Comparing 〈*P*_2_〉_SAXS_ and 〈*P*_2_〉_PLI_ in Figure 3b it is evident that the onset of the Maltese‐cross in PLI, is detected at shear rates corresponding to virtually constant order parameter. This is below the critical shear rate for transition to region (II), when a pronounced increase in the order parameter is detected, ${\dot{\gamma }}_{\mathrm{orien}}^{\mathrm{PLI}}<{\dot{\gamma }}_{\mathrm{orien}}^{SAXS}={\dot{\gamma }}_{cr}^{\left(II)-(III\right)}$, meaning that orientation at mesoscale in the hierarchy occurs before there is a progressive orientation in the flow direction at nanoscale.

Another example is presented in Figure 3c in the form of a surface modified 2.5% [P]‐CNC. In this example, azetidinium salts have been conjugated to the sulfate groups on the surface of the CNCs into 2.5% [P]‐CNC‐OH‐2‐Prop‐C_2_‐N‐C_2_, which in previous investigations was shown to be disruptive of self‐assembly at rest (no flow).^[^ ^23^, ^32^, ^33^ ^]^ This is supported by the significant increase in viscosity magnitude compared to the reference suspension in Figure 3a. The grafting of chains on the surface of CNCs causes an increase in effective CNC size and promotes agglomerates that can be disrupted by shear to induce birefringence.^[^ ^32^ ^]^ Thus, the system starts from initial conditions with no hierarchical order/alignment, i.e., 〈*P*_2_〉_SAXS_ ≈ 0 and *P*_2 PLI_ ≈ 0. Once again, orientation at mesoscale, ${\dot{\gamma }}_{\mathrm{orien}}^{\mathrm{PLI}}$, can be identified as a Maltese‐cross pattern before the onset of any significant orientation at nanoscale ${\dot{\gamma }}_{\mathrm{orien}}^{SAXS}$.

In contrast to the two examples outlined above, the [P]‐CNC in Figure 3d of the same 2.5% CNC concentration but with with primary nanoparticles of predominantly individual CNCs as building blocks for liquid crystalline orders (or conversely the absence of aggregates/bundles or significant agglomerates), shows that the onset of orientation in the flow direction at nanoscale is simultaneous across the lengthscales probed. This corresponds to the transition between regions (I)–(II), i.e., ${\dot{\gamma }}_{cr}^{SAXS}={\dot{\gamma }}_{cr}^{\mathrm{PLI}}={\dot{\gamma }}_{cr}^{\left(I)\to (II\right)}$. In addition, ${\dot{\gamma }}_{cr}^{\left(II)\to (III\right)}$ appears to also correspond to a pronounced increase in birefringence for a simultaneous multiscale orientation.

To further emphasize the significance of these results, we can consider the shear rate distribution along the beam path length, see also Section S3.1 (Supporting Information). In a parallel‐plate Rheo‐PLI‐SAXS experiment, for constant angular velocity there is a linear shear rate distribution from zero at the rotation axis to a maximum value at the edge of the moving geometry (rigid body rotation). Thus, while all data are reported at the nominal (or effective) shear rate of the rheological experiment, PLI data are taken at the exact radial position where the local shear rate equals the nominal shear rate, while SAXS data probes the existence of any anisotropy at all shear rates along the beam path. Based on this a discrepancy between the onset of SAXS and PLI orientation would be expected simply considering the shear rate distribution in the measuring geometry, $\dot{\gamma }\left(r\right)$. Because ${\dot{\gamma }}_{max}=3\dot{\gamma }/2$, where ${\dot{\gamma }}_{max}$ is the maximum shear rate at the edge of the rotating plate, *r* = *R*, it would be expected that orientation at nanoscale would be detected at shear rates before it occurs at the nominal shear rate in PLI. However, in Figure 3b,c orientation in SAXS is observed only after mesoscale orientation is detected in PLI in the form of the Maltese‐cross pattern, ${\dot{\gamma }}_{\mathrm{orien}}^{SAXS}>{\dot{\gamma }}_{\mathrm{orien}}^{\mathrm{PLI}}$.

The above observation raises the question of how ${\langle P_{2}\rangle }_{SAXS}\left(\dot{\gamma }\right)$ data in the parallel‐plate Rheo‐PLI‐SAXS experiments would compare with other measuring geometries. Thus, we have compared parallel‐plate Rheo‐SAXS data with concentric cylinder measurements, Figure S11 (Supporting Information). The data implies that, at least based on the cases which exhibit a 3‐region behavior, there is little to no difference between the measuring geometries in terms of the critical shear rates associated to the transitions between the regions. However, it is important to briefly highlight that the evolution of the order parameter can differ significantly. Comparing the order parameters for the same sample in Figure S11b (Supporting Information) we observe in the concentric cylinder geometry data a similar trend as previously reported.^[^ ^13^ ^]^ Therefore, despite the considerable difference in the order parameter magnitude, we are essentially probing the same or similar representative microstructural dynamics. Moreover, a perhaps over‐simplified expectation would be that the onset of birefringence should coincide with or be the result of an increase in nanoscale orientation towards creating the long‐range alignment conditions for the formation of uniformly oriented optical indicatrixes. However, in this case, while there is a fraction of nanoscale alignment in the flow direction, 〈*P*_2_〉_SAXS_ is relatively small and remains constant up to ${\dot{\gamma }}_{\mathrm{orien}}^{\mathrm{SAXS}}$. We associate the nanoscale orientation to likely correspond to nanoparticles contained within the mesoscale and not to the isotropic phase, orientation of which has been attributed to the third region.^[^ ^13^ ^]^ The absence of birefringence at the observation lengthscale chosen does not mean the total absence of birefringence. Thus, while we are not interrogating all the lengthscales in‐between PLI and SAXS, it is clear that there is a transfer of orientation (and likely deformation / elongation) from mesoscale, which is observed first, to the nanostructure within the mesodomains.

Overall, for the biphasic [B]‐CNC suspensions, where the nanostructure appears to contain interconnected bundles (TEM), small tactoids can be identified in POM but they are surrounded by considerable agglomerates, Figure S7a (Supporting Information). In comparison, [P]‐CNC, where the nanostructure contains predominantly individual CNC crystallites, clear tactoids and larger domains having a variety of sizes and shapes ca be clearly identified in POM, Figure S7b (Supporting Information). Furthermore, [P]‐CNC suspensions if slightly perturbed by flow (e.g., immediately after pouring on the microscopy slide), clearly show the presence of highly elongated chiral nematic domains in POM, where their optical axis is perpendicular to the alignment direction of the domains, Figure S7c (Supporting Information). Such a relative orientation between **m** and **n**_*m*_ means that **n**_*p*_ and **n**_*m*_ ensure that nano‐meso orientation in the flow direction appears to develop at the same time, in agreement with previous observations,^[^ ^14^ ^]^ see also the schematic explanation in Figure S14a (Supporting Information). It can be therefore inferred, that for [B]‐CNC suspensions the long range assembly is hindered by the presence of agglomerates. The same seems to be valid if [P]‐CNC suspensions with surface grafted chains, which promote agglomerates and hinder self‐assembly. In such cases, the onset of preferential alignment in the flow direction at mesoscale occurs before an increase in nanoscale preferential alignment can be detected. This could evidence that the orientation and distortion of such unstructured or partially structured domains/agglomerates leads to the creation of domains oriented in the flow direction with effective birefringent properties (effective refractive indices), Figure S14b (Supporting Information). Notably, this is valid for [B]‐CNC suspensions even when lowering their concentration such that their shear viscosity is comparable to that of 2.5% [P]‐CNC, see Figure S13b (Supporting Information).

### 2.3 Liquid–Crystalline Systems

**Figure 4:**
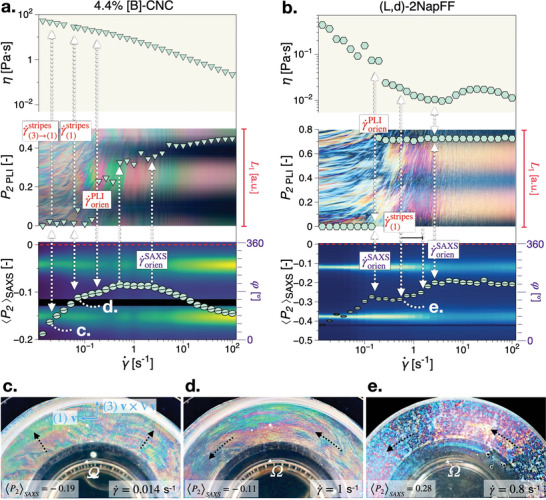
Multiscale analysis (see Figure 2a) of concentrated liquid crystalline phase of: a) 4.4% [B]‐CNC; b) (L,d)‐2NapFF. c–e) PLI still frame visualizations corresponding to the datapoints marked in (a,b).

Asynchronous multiscale orientation can also be observed for liquid‐crystalline phase systems, i.e., assembly systems without an isotropic phase, Figure 1b. In contrast to the biphasic systems, the onset of the Maltese‐cross pattern corresponds to a material process of progressive loss of preferential orientation at nanoscale. In **Figure** **4** we compare a [B]‐CNC with a liquid crystalline low molecular weight gelator system, (L,d)‐2NapFF, to form the basis of a more general interpretation of the results. We note that concentrated suspensions are significantly influenced by the initial conditions at the beginning of shear, in the absence of a complete sample relaxation, and the onset of complex mesoscale director fields. By initial conditions we refer to the initial director field as a result of setting the measuring position. Although CNCs are chiral‐nematic forming systems, due to the relatively high concentration (physical confinement), the 4.4% [B]‐CNC does not form a cholesteric phase within a reasonable experimental timescale. The low molecular weight gelator investigated comprise thin‐wall tubular micellar structures formed by the self‐assembly of a functionalized dipeptide molecule, (L,d)‐2NapFF. While the liquid crystalline properties of the (L,d)‐2NapFF have yet to be throughly understood, their aspect ratios have been shown to be in excess of 70 based on cryo‐TEM and small‐angle neutron scattering.^[^ ^34^, ^35^ ^]^ In addition, polarized light microscopy of the tested samples revealed no “fingerprint” textures characteristic of chiral nematic structures, see Figure S5 (Supporting Information). Both the 4.4% [B]‐CNC in Figure 4a and (L,d)‐2NapFF in Figure 4b share a high level of initial orientation followed by a progressive loss of preferential orientation at nanoscale (increase in 〈*P*_2_〉) with increasing shear rate. The magnitude of the order parameter at the onset of shear suggests that overall a substantial amount of nematic domains have remained preoriented in the plane of shear after the waiting time prior to the starting of the tests.

**Figure 5:**
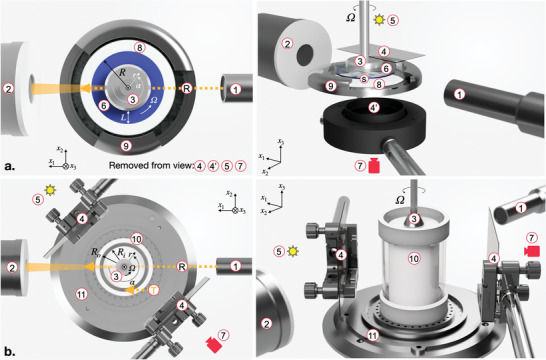
Schematic of the Rheo‐PLI‐SAXS custom experimental setups: a) using a parallel‐plate and b) a concentric‐cylinders measuring geometries. Notations: (R) – radial incident X‐rays, (T) ‐ tangential incident X‐rays, (1) – X‐ray source, (2) – detector tube, (3) – steel part of the measuring geometries (4,4') – polarizer / analyzer, (5) – LED light source, (6) – transparent part of the upper plate measurement geometry, (7) – DSLR camera, (8) – glass bottom plate, (9) – custom bottom plate holder, (10) – outer cylinder, (11) – concentric cylinder setup mounting system; (s) – sample (not included in the bottom row to preserve clarity).

Although beyond the scope of the present study, we note that the loss of preferential orientation can be associated with (i) a transition zone between the gap‐setting (squeeze flow) induced structure to a restructuring in the azimuthal (shear flow) direction and (ii) the appearance of flow‐induced PLI textures in the form of stripes. The formation of textures in the form of stripes is a rather common occurrence in the flow of liquid crystalline systems signalling the presence of secondary flows (rolls) in the flow. They have been observed in nematic phase forming polymeric systems and lamellar nanostructured systems.^[^ ^8^, ^36^, ^37^, ^38^, ^39^, ^40^ ^]^ In CNC suspensions they have been evidenced using microscopy slides and in steady shear flow using a similar Rheo‐optical system.^[^ ^22^, ^23^ ^]^ Interestingly, (i) and (ii) coincide for 4.4% [B]‐CNC, where radially‐induced stripes, Figure 4c, transition above ${\dot{\gamma }}_{\mathrm{stripes}}^{\left(3)-(1\right)}$ (note: the subsript notation refers to the orientation of stripes in the vorticity direction (3) and velocity direction (1) after the onset of shear) with increasing shear rate into azimuthal stripes above ${\dot{\gamma }}_{\mathrm{stripes}}^{\left(1\right)}$, Figure 4d. This is further supported by Rheo‐PLI‐SAXS experiments we have performed using a custom concentric cylinder setup, see **Figure** **5**, Figure S15 and S16 and description thereof in Section S5.2 (Supporting Information). In contrast, for (L,d)‐2NapFF (i) and (ii) are distinct with azimuthal stripes observable only above ${\dot{\gamma }}_{\mathrm{stripes}}^{\left(1\right)}$, Figure 4e.

However, most importantly for the present study is that the onset of the Maltese‐cross pattern, ${\dot{\gamma }}_{\mathrm{orien}}^{\mathrm{PLI}}$, is detected during the loss of preferential orientation at nanoscale and approximately one decade in shear rate before an increase in preferential orientation in the flow direction at nanoscale (decrease in 〈*P*_2_〉) can be inferred for 4.4% [B]‐CNC. Similar results have been obtained for a suite of CNCs with different counterions (data not shown). Interestingly, in (L,d)‐2NapFF a slight increase of nanoscale preferential alignment (decrease in 〈*P*_2_〉) can be detected both at the onset of the Maltese‐cross pattern in PLI (${\dot{\gamma }}^{{\mathrm{PLI}}_{\mathrm{orien}}}$) until the onset of stripe textures (${\dot{\gamma }}^{{\mathrm{stripes}}_{(}1)}$) as well as approximately one decade higher, similarly to 4.4 [B]‐CNC. However, the similarity ends here, as (L,d)‐2NapFF shows a local shear thickening behavior. We note that beyond the main theme of asynchronous nano‐micro hierarchical orientation, all other experimental features outlined serve only as an exciting outlook into what further new insights could be gained through the newly developed Rheo‐PLI‐SAXS setup.

## 3 Conclusions and Outlook

Multiscale orientation analysis based on a novel combination of simultaneous rheological measurements, polarized light imaging and small‐angle X‐ray scattering, Rheo‐PLI‐SAXS, reveals a suite of unique intriguing insights into the multiscale behavior of hierarchical self‐assembling nanostructured fluids. A particular feature in the observations made insofar, is the predominant discrepancy between microscale orientation, as revealed by the Maltese‐cross pattern in PLI, and orientation at nanoscale through SAXS. Note that we attribute here nanoscale alignment to the orientation inside the mesoscale. Therefore, a crucial factor determining the nano‐micro alignment propagation inside the mesophase appears to be the extent of confinement effects induced by the elongation and breakup of mesophase domains. Knowing the critical shear rates for multiscale assembly could be used for achieving an unprecedented level of control of the hierarchical assembly in e.g. coating or film forming shear dominated forming operations. In biphasic systems, for example, the possibility of controlling the orientation of the cholesteric axis relative to that of the tactoids by orienting the mesoscale without disrupting the nanoscale orientation inside the mesoscale becomes attainable. From a fundamental point of view, the nano‐ meso‐orientation decoupling described, while based on a rather broad spectrum of systems, will need a closer inspection especially in terms of the role of CNC surface modifications. Furthermore, beyond the cases studies presented, there are numerous classes of systems^[^ ^41^ ^]^ that could be investigated with the technique which could yield unique insights into their multiscale orientation and structuring, such as nanoparticles of other morphologies, e.g., 2D,^[^ ^41^ ^]^ hybrids thereof,^[^ ^42^ ^]^ systems with different packing phases,^[^ ^43^ ^]^ surfactant solutions, etc. Furthermore, besides the steady shear measurements presented, there are other rheological tests that could be further explored.^[^ ^44^, ^45^, ^46^ ^]^ Furthermore, the PLI could be extended towards more advanced optical techniques, such as the use of high‐speed polarization cameras,^[^ ^47^ ^]^ some of which we are currently developing. If performed in reflection mode, for example, a single rotation of the upper plate would yield a complete state of stress over the entire range of the shear rate distribution inside the flow domain using the stress‐optical rule.^[^ ^48^ ^]^ Taking a broader perspective, from a materials science point of view, such insights contribute towards a precise combination of surface modification, transport/processing and assembly into products for bio‐based hierarchical nanomaterials.

## 4 Experimental Section

### 4.1 Materials

A list of the samples investigated and some of their main characteristics of relevance to the study is presented in **Table** **1** below.

**Table 1:** List of the samples discussed. Abbreviations: CNC ‐ cellulose nanocrystals, [B] ‐ bath sonicated; [P] ‐ probe sonicated; (L,d)‐2NapFF ‐ thin‐wall nanotubes; I ‐ isotropic phase; N ‐ nematic phase; N* ‐ chiral nematice phase.

Sample	ϕ	Phase	$\eta (\dot{\gamma })$	Figure
[B]‐CNC	1.9%	I	3‐region viscosity	S13.b
	2.5%	I/N*	3‐region viscosity	3.a
	3.9%	N*/N	3‐region viscosity	3.a
	4.4%	N*/N	η_0_, shear thinning	4.a,b
[P]‐CNC	1.2%	I/N*	Weakly shear thinning	S13.a
	2.5%	I/N*	3‐region viscosity	3.c
[P]‐CNC‐OH‐2‐Prop‐C_2_‐N‐C_2_	2.5%	—	η_0_, shear thinning	3.b
[B]‐CNC‐OSO_3_‐TEOA	2.5%	—	η_0_, shear thinning	S13.c
(L,d)‐2NapFF	10 mg mL^−1^	N	shear thinning, thickening	4.c

#### 4.1.1 Cellulose Nanocrystal (CNC) Suspensions

CNC powder purchased from CelluForce (Montreal, Canada) was added and mixed in Milli‐Q water for preparing [B] and [P] CNC suspensions. For [B], the suspensions were sonicated using an ultrasound bath for one hour and subsequently, a bench shaker was used for mixing and homogenizing the suspension for 72 h. Based on previous studies,^[^ ^31^ ^]^ we estimate the aspect ratio of [B]‐CNCs to be between 25 and 75 from atomic force microscopy (AFM) analysis, with diameters and lengths approximately 4 nm and 100–300 nm, respectively. Further details and characterization of similar suspensions, such as AFM, ζ‐potential, cryo‐TEM etc. can be found elsewhere.^[^ ^21^, ^23^, ^45^ ^]^ The preparation procedure for [P]‐CNCs has been chosen based on the study by Parton et al.^[^ ^7^ ^]^ to access the primary CNC crystallites by probe‐sonicated to the equivalent of 15 kJ mL^−1^. As [B]‐CNC have been bath‐sonicated and not probe sonicated, we consider that the sonication dose tends to 0 kJ mL^−1^ in the data of Parton et al.^[^ ^7^ ^]^ This is because probe sonication ensures a uniform and focused transmission of ultrasound waves, and can outperform bath sonication by a factor of 1000.^[^ ^49^ ^]^ Thus, [B]‐CNC suspensions are predominantly composed of CNC aggregates and bundles while [P]‐CNC suspensions are predominantly composed of primary CNC particles. During probe‐sonication the (mean effective 3D) aspect ratio of the CNCs has been shown to reduce by approximately 25%. This is confirmed by TEM and POM, see Figure S7 in Section S5 (Supporting Information).

#### 4.1.2 Surface Grafted CNC Suspensions

The surface modification follows the procedure of Wojno et al.^[^ ^23^ ^]^ A 2.5% CNC suspension was prepared by mixing CelluForce CNC powder (4g) in DI water (96 mL) using a magnetic stirrer for 2 h. After homogenous mixing, the cloudy mixture of CNC was sonicated (6.25 J mL^−1^) for 10 mins, (500 W, 40% Amplitude, 20 KHz, Ultrasonic Processor VC505 ‐1/2’’ microtip) to obtain a clear solution. Further, dialkyl (diethyl) azetidinium salt (C_2_‐N‐C_2_‐Prop‐2‐OH or 1‐ethyl‐3‐hydroxy‐1‐ethyl azetidin‐1‐ium chloride) was added in 1:1 mole equivalent to sulfate groups present on the surfaces of cellulose nanocrystals as described in our previous report. The mixture was vigorously stirred and heated for 4 h at 90 °C. After the reaction, the suspension was subjected to dialysis to remove unreacted salt and other impurities. After purification, the suspension was stored in the refrigerator at 4 °C prior to testing.

#### 4.1.3 Suspension of Thin‐Wall Nanotubes, (L,d)‐2NapFF

Stock solutions of gelator (L,d)‐2NapFF were prepared at a final concentration of 20 mg mL^−1^. This was done by suspending 200 mg of gelator in deionised water and one equivalent of sodium hydroxide solution (0.1 M) to a total volume of 10 mL. The solutions were stirred at 1000 rpm overnight in a falcon tube to allow complete dissolution of the gelator. The pH of the solution was then adjusted to pH 11 ± 0.1 by using 1M NaOH. (L,d)‐2NapFF is a low molecular weight gelator (LMWG) that self‐assembles into thin‐wall nanotubes at high pH.^[^ ^35^ ^]^ The high‐resolution structure of the nanotubes at high pH has recently been reported.^[^ ^34^ ^]^ The molecules were shown to pack through non‐covalent interactions (mainly π − π stacking) in a left‐handed helical manner to form the large hollow structures in solution. Further details about their chemical structure can be found elsewhere.^[^ ^50^, ^51^, ^52^ ^]^ They have previously shown to exhibit liquid‐crystalline textures in Rheo‐PLI investigations and shear‐induced Maltese‐cross patterns.^[^ ^50^ ^]^ While not yet fully determined, based on previous cryo‐TEM images and small‐angle neutron scattering data, (L,d)‐2NapFF can be estimated to have aspect ratios in excess of 70^[^ ^34^, ^35^ ^]^ and have shown no evidence of “fingerprint” textures in POM, see Figure S5 (Supporting Information). A consistent diameter of 30 nm was determined from cryo‐TEM and small‐angle neutron scattering (SANS),^[^ ^34^ ^]^ however, the lengths and their variability could not be determined as they exceeded the typical observation lenghtscales of the respective techniques.

### 4.2 Methods

An schematic overview of the two Rheo‐PLI‐SAXS experimental setups is shown in Figure 5, with each unit described separately.

#### 4.2.1 Rheology

All rheological experiments were performed on an Anton Paar MCR702 Multidrive rheometer in single motor‐transducer configuration. Three types of measuring geometries were used: (i) a glass parallel plate 43 mm in diameter (ii) a 50 mm steel cone‐plate and (iii) a concentric cylinders standard SAXS polycarbonate geometry with an inner cylinder radius of 24.5 mm and an outer cup of 25 mm outer (radius ratio: 0.98), see Section S3.1 (Supporting Information). The main Rheo‐PLI‐SAXS setup is based on (i) while tests have also been performed on a custom version of (iii).

The novel parallel‐plate Rheo‐PLI‐SAXS is based on a modified Peltier measuring cell to allow for X‐Ray access, see Figure 5a. The steady shear measurements were performed using a custom steady state procedure elaborated in several of our previous studies.^[^ ^12^, ^23^, ^45^ ^]^ The procedure includes an optimized transient time to steady state for each measured point. All transient data was recorded and steady‐state values were subsequently evaluated. The shear rate was varied between 0.014 and 100 s^−1^ in parallel‐plate tests and between 0.0014 and 400 s^−1^ in some concentric cylinder tests.

#### 4.2.2 Polarized Light Imaging (PLI)

The PLI setup is an adaptation of a custom transmission setup previously used for investigating birefringent suspensions in flow.^[^ ^21^, ^23^, ^45^, ^53^ ^]^ The PLI optical train for the plate‐plate measuring geometry consisted of two linear polarizers (Edmund Optics, Barrington, USA) (i), Figure 5. For concentric cylinders, a combination of linear and circular polarizer was used. PLI was acquired in the form of video recordings (1920×1080 pixel) from which space‐time diagrams were constructed.^[^ ^22^ ^]^ Still frame extracts at selected shear rates for all the multiscale data discussed can be found in Figures S4 and S5 (Supporting Information). The imaging data was converted into the CIELab colorspace and the Euclidean norm was used in Equation (1) instead of *I* as

(1) $$I_{im}=\sqrt{L^{*2}+a^{*2}+b^{*2}}$$

where L* is the luminance and a* and b* are the chroma, see Figure S2 (Supporting Information) for a schematic illustration of the color space. Examples of PLI still frame, space‐time diagram and *I*_*im*_ variation with shear rate are shown in Figure 2.d,e,f for 2.5% [B]‐CNC.

#### 4.2.3 Small‐Angle X‐Ray Scattering (SAXS)

The SAXS experiments were performed at the CoSAXS^[^ ^54^ ^]^ and ForMAX beamlines,^[^ ^55^ ^]^ MAX IV Laboratory. At CoSAXS, we employed an X‐ray beam energy of 15 keV and a beam size at the sample position of ≈62 × 34 µm^2^ (horizontal × vertical), while data were collected 3.5 m downstream of the sample using an EIGER2 X 4M photon‐counting pixel detector.^[^ ^56^ ^]^ At ForMAX, the X‐ray beam energy was set to 20.2 keV, the beam size at the sample position to ≈300 × 300 µm^2^, and an EIGER2 X 4M detector was placed 3.1 m downstream of the sample.

Example scattering patterns for the (L,d)‐2NapFF are shown in Figure 2a. Hermans orientation parameter from azimuthal integration of the scattering patterns was determined as

(2) $${\langle P_{2}\rangle }_{SAXS}=\frac{\int_{0}^{\pi }\frac{1}{2}\left(3\cos^{2}\varphi -1\right)I{\left(\varphi \right)}_{q}\sin\varphi d\varphi }{\int_{0}^{\pi }I{\left(\varphi \right)}_{q}\sin\varphi d\varphi }$$

where φ is the azimuthal angle of the scattering pattern, see Figure 2a and *I*(φ)_*q*_ is the scattering intensity in the integrated *q*‐range. Examples of azimuthal integrations as color maps and as dataplots are shown in Figure 2b,c for 4.4% [B]‐CNC. Within the [0, π] integration limits used in Equation (2), 〈*P*_2_〉_*SAXS*_ ∈ [− 0.5, 1], where 〈*P*_2_〉_*SAXS*_ = 0 signifies random orientation of nanoparticles 〈*P*_2_〉_*SAXS*_ = 1 a fully oriented nanostructure with a preferential orientation perpendicular to the flow direction, and 〈*P*_2_〉_*SAXS*_ = −0.5 signifying a fully oriented nanostructure with a preferential orientation in the flow direction. The integration *q*‐range was chosen based on radial integration of the scattering patterns, see Section S4 and especially Figure S3 (Supporting Information). All azimuthal integrations reported in the manuscript correspond to region around the structural peak identified in the radial integration, see *q*_2_ in Figure S3 (Supporting Information). For comparison, two other integration intervals were considered for lower and higher *q*‐ranges relative to the structural peak (*q*_1_ and *q*_3_). Since concentration and to a limited extent shear influences the structural peak, see Figure S3b,c (Supporting Information), the integration *q*‐range is not the same throughout the samples. A list of *q*‐ranges used can be found in Table S1 (Supporting Information). In parallel‐plate flow, the beam was positioned in the middle of the measuring gap (*h*/2).

#### 4.2.4 Experimental Synchronization

A waiting time of approximately 3 min was kept between setting the sample to the measuring gap and beginning of the Rheo‐PLI‐SAXS experiments. No pre‐shear has been applied prior to the measurements and the steady shear tests were performed only from low to high shear rates. The optical camera (PLI) was started using a remote control software around the time of the start of the rheological experiment, with the data further corrected at data analysis. A custom macro was written to acquire the SAXS data and it was at first timed and in latter experiments triggered such that for each viscosity measuring point SAXS data was acquired for 5 times with 1 s of exposure time (PP and CP geometries) and 2 times with 0.1 s of exposure time (CC geometry) when the transient data (monitored live) was at steady state.

POM and TEM have been used to assess the multiscale structure of the samples at rest, see Section S5 (Supporting Information) for further details.

## Conflict of Interest

The authors declare no conflict of interests.

## Author Contributions

R.G., A.T., M.B. and K.S. equally contributed to the work. R.G. contributed to the investigation, writing, and review/editing of the manuscript. A.T. was responsible for software development, formal analysis, data curation, supervision, and conceptualization, as well as project administration and writing/reviewing the manuscript. S.W. was involved in investigation, visualization, and writing/reviewing the manuscript. M.B. handled formal analysis, data curation, software, visualization and writing/reviewing the manuscript. K.S. contributed to the investigation, visualization, and writing/reviewing the manuscript. A.K.S. took part in investigation and writing the original draft, in addition to reviewing and editing the manuscript. K.N. worked on programming, data curation, project administration, formal analysis, supervision, conceptualization, and writing/reviewing the manuscript. V.G. was involved in investigation, visualization, and writing/reviewing the manuscript. S.B. contributed to investigation, writing the original draft, and reviewing/editing the manuscript. M.L. provided supervision, funding acquisition, and writing/reviewing the manuscript. A.M. was involved in funding acquisition, and writing/reviewing the manuscript. G.W. contributed to supervision and writing/reviewing the manuscript. T.N. contributed to supervision, resources, project administration, funding acquisition, and writing/reviewing the manuscript. R.K. contributed to conceptualization, methodology, investigation, formal analysis, visualization, data curation, software, supervision, project administration, funding acquisition, resources, and writing the manuscript and review/editing.

## Supporting information

Supporting Information

## Data Availability

The data that support the findings of this study are available from the corresponding author upon reasonable request.
