# Supplementary material for: Propagation of Orientation Across Lengthscales in Sheared Self‐Assembling Hierarchical Suspensions via Rheo‐PLI‐SAXS
Source: Adv Sci (Weinh). 2024 Dec 25;12(7):2410920. doi: 10.1002/advs.202410920 (PMC11831526; doi:10.1002/advs.202410920)
Supplement: Supplementary file 1 — Supporting Information [file ADVS-12-2410920-s001.pdf]

ADVANCED SCIENCE
Open Access

Supporting Information

for *Adv. Sci.*, DOI 10.1002/advs.202410920

Propagation of Orientation Across Lengthscales in Sheared Self-Assembling Hierarchical Suspensions via Rheo-PLI-SAXS

*Reza Ghanbari, Ann Terry, Sylwia Wojno, Marko Bek, Kesavan Sekar, Amit Kumar Sonker, Kim Nygård, Viney Ghai, Simona Bianco, Marianne Liebi, Aleksandar Matic, Gunnar Westman, Tiina Nypelö and Roland Kádár**

Supporting Information

SI 3 Lengthscales

The matter of material hierarchy and multiscale analysis can be generalized using a relative lengthscale

$$K = \frac{\mathscr{L}}{\mathscr{L}_{ref}} \tag{S3}$$

where $\mathscr{L}$ is any relevant characteristic material of characterization method lengthscale and $\mathscr{L}_{ref}$ is a reference lengthscale, which we consider here as a dimension of the primary nanoparticle. Several lengthscales to consider are:

- $\mathscr{L}$, a characteristic material lengthscale. This can refer to any of the hierarchical structures in Fig. 1.a.
- $\mathscr{L}_{exp}$ that refers to the dimensions at which a characterization technique interrogates a material structure, and
- $\mathscr{L}_{obs}$, the extent of the observation domain of a characterization technique.

Subscript *exp* is used in the following to distinguish K when referring to experiments that probe the hierarchy, which we define using $\mathscr{L}_{exp}$ in Eq. (S3) as a characteristic lengthscale probed by a certain technique. From a materials characterization point of view, the larger K_{exp}, the more difficult it is to separate the contributions of the hierarchy with $K < K_{exp}$. Meanwhile, experiments performed for $K_{exp} \approx 1$ obscure the higher orders of the hierarchy. It is important to note that the material lengthscales probed could be averaged over a larger lengthscale, $\mathscr{L}_{obs}$. Thus, because in principle e.g. for parallel-plate measuring geometries (PP) $\mathscr{L}_{obs} \propto 2 \cdot R$ the observation lengthscales are the same for Rheology, PLI and SAXS (pathlength). This observation is relevant to emphasize that the three techniques capture the same shear rate distribution. A more consistent distinction between the methods in terms of observation lengthscales is whether or not the output is averaged or not over $\mathscr{L}_{obs}$. To compare different techniques it could also be perhaps more suitable to refer to characteristic observation volumes. In terms of $\mathscr{L}_{exp}$ SAXS interacts with matter in the range nanoscale range, PLI is susceptible to lengthscales that cover nanoscale and mesoscale and Rheology includes also 'macroscale', by which here we refer to the onset of flow instabilities (collective motion in Fig. 1.a). Importantly, SAXS and Rheology average all interactions over their respective observation volumes while PLI is an average over the polydomain texture in the measuring gap. The challenge for the multiscale analysis of such systems is not only that a simultaneous multiscale experiment needs to be susceptible to comparable to the hierarchy levels, $\mathscr{L}_{exp} \sim \mathscr{L}$, it can be useful if the observation lengthscale $\mathscr{L}_{obs} >> \mathscr{L}_{exp}$ and that the output is not averaged over the observation domain. The latter makes PLI crucial to identify other possible specific long-range flow features such as e.g. instabilities, Fig. 1.a, or simply experimental artefacts such as bubbles, sample inhomogeneities or incomplete filling of the measuring gap. We estimate that in the PLI system used the smallest structures that could be identified directly from the visualizations (without the need for polarized light) to be $\approx 50\ \mu$m.

By choosing the primary nanoparticle hierarchical level as $\mathscr{L}_{ref}$, we have effectively neglected from the description of the hierarchy the molecular lengthscales inside the CNCs as well as the lengthscales associated to the particle-solvent and particle-particle interactions. The latter are however considered indirectly through the modified CNC suspensions.

Birefringence at flow scale is an average over the measuring gap of optical anisotropic contributions sufficiently smaller than the observation lengthscale:

$$\Delta n = \Delta n_{np} + \Delta n_{meso} + \Delta n_{orien}. \tag{S4}$$

While Δn_{np} can be neglected at such high observation lengthscales (the relevance of it is that CNC is itself crystalline), $\Delta n_{orientation}$ can be a result both of the liquid crystalline domains (assembly) as well as the individual nanoparticles in the case of monodomain paranematic flow. On the other hand, at rest, (no flow) if any birefringence is observable, then it can be attributed to liquid crystalline domains, Δn_{meso}.

SI 3.1 Measuring geometries

Viscometric flows can be generated using a variety of so-called measuring geometries on a rotational rheometer. In all cases, the velocity and shear stress distribution in the measuring gap are solutions of the Navier-Stokes equations, expressed in cylindrical coordinates, for incompressible flows. Historically the most significant and also the most practical for Rheo-SAXS measurements, the flow between concentric cylinders (CC), Fig. S1, has been firstly elaborated in Newton's *Philosophiæ Naturalis Principia Mathematica*. The velocity distribution for the general case of independently rotating cylinders is

$$v(r) = Ar - \frac{B}{2r} \tag{S5}$$

where r is the radial direction of a coordinate system associated to the measuring geometry and A, B are

$$A = 2\Omega \frac{R^2 - R_1^2}{R^3 - R_1^2} \tag{S6}$$

$$B = \Omega \frac{R^2}{R^2 - R_1^2} \tag{S7}$$

hus, the velocity profile can be approximated as linear only for radius ratios, R/R_1, as close as possible to unity. In such a case, the shear rate in the measuring gap is

$$\dot{\gamma} = \frac{R\Omega}{h} \tag{S8}$$

where h is the gap between the two cylinders. SAXS measurements in the radial incident x-ray configuration (R) thus capture a uniform flow field in the velocity - vorticity plane, i.e. (1)-(3) or $\mathbf{v}$ - $\nabla \times \mathbf{v}$ and in tangential configuration (T) in the velocity gradient - vorticity plane, i.e. (2)-(3) or $\nabla\mathbf{v}$ - $\nabla \times \mathbf{v}$.

Parallel-plate (PP) measuring geometries, Fig. S1.b are ubiquitous especially for samples with high viscosities or for most rheological hyphenated techniques. The velocity distribution along a shear plane of constant radius r is linear, however, since the flow is driven by the upper plate which is in rigid body rotation, the shear rate is a function of the radial coordinate r as

$$\dot{\gamma}(r) = \frac{\Omega r}{h}. \tag{S9}$$

Thus a nominal shear rate is defined as control parameter in PP flows at $r = 2/3R$, where R is the radius of the moving plate, i.e. $\dot{\gamma} = 2/3(R\Omega/h)$. Thus, in (R) configuration, technically the (1)-(2) plane is probed, and in (T) the (2)-(3) plane. However, in both cases the incident beam is interacting with a range of shear rates and possibly states. In (R) this is from a maximum at $r = R$ (note: $\dot{\gamma}_{max} > \dot{\gamma}$) to effectively zero in the center of the geometry. However, parallel-plate geometries are very easy to probe with complementary methods in the (1)-(3) plane. Furthermore, when operated in reflection mode, effectively at steady state following the analysis of an instantaneous say imaging snapshot a full set of states corresponding to a steady shear test can be extracted as function of the radius. This can be particularly useful when applying e.g. more quantitative PLI techniques such as the stress-optical rule. To summarize, while PP measuring geometries present some disadvantages for SAXS measurements they also have significant advantages for coupled techniques the full potential of which have not been reached in this study.

An alternative to parallel-plate geometries are cone-plate (CP) measuring geometries, Fig. S1.c., where because the gap height is a function of the radius, $h = h(r)$ a uniform shear rate is obtained across the radius

$$\dot{\gamma} = \frac{\Omega}{\beta} \tag{S10}$$

where β is the angle of the cone. From SAXS point of view, however, measurements in (R) cannot be performed due to the very narrow (truncated) gap in the middle making only (T) configurations experiments possible. Furthermore, in many cases, fillers can jam in the narrow center gap thus causing artefacts in the measurements. In the case of CNC suspensions PP and CP measurements have been compared with negligible differences found[20].

Interestingly, as seen further in Section S11, the main difference between the three measurement setups in terms of SAXS data appears to be the magnitude of the order parameter, with CC recording the highest followed by CP and CC. It needs to be underlined however, that at least based on the order parameter critical shear rates for transition between the three-regions, PP, CP and PP all probe the same structural transitions at approximately identical shear rates.

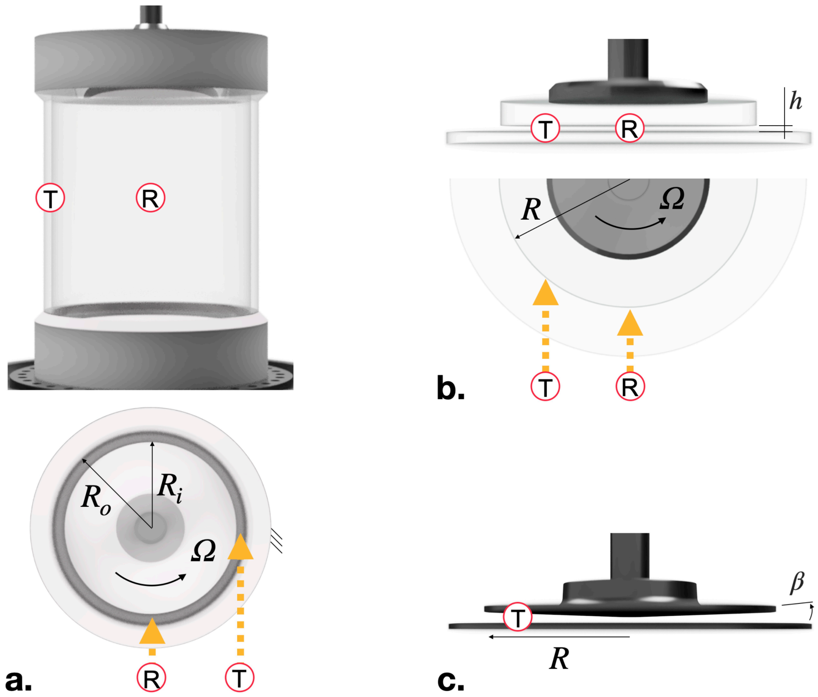

Figure S1. Schematics of the measuring geometries: **a**. concentric cylinders, **b**. parallel-plate and **c**. cone-plate. Ω is the angular velocity of the rotating part.

SI 4 Complements to data analysis

An illustration of the CIELab colorspace is presented in Fig. S2.

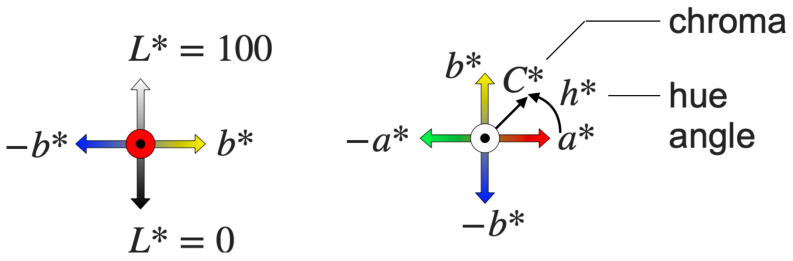

Figure S2. Orthogonal projections of the CIELab colorspace used in the PLI image analysis.

Radial integrations of the SAXS scattering patterns are compared in Fig. S3. Note that the q-range selection was done based on linear scattering plots while in the figure they are represented in double-log.

A list of q-range binning for all the samples analyzed can be found in Table S1. The structural peak range, q_2, was used in all the PP data presented as multiscale analysis in this work. In a preliminary set of tests, we had previously observed that in most cases the shear rate difference between the PLI and SAXS detection of orientation is at its minimum at q_2.

In Fig. 3.a we have illustrated how the order parameter plots for SAXS were constructed:

$$D_1 = 2 \cdot (1 - |\langle P_2 \rangle|) \quad \text{(S11)}$$

$$D_2 = 4 \cdot (0.5 - |\langle P_2 \rangle) \quad \text{(S12)}$$

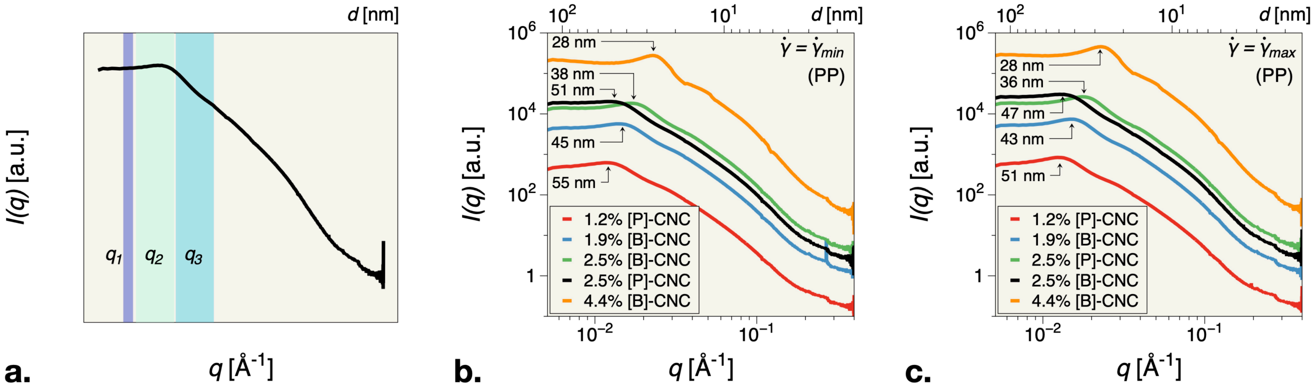

Figure S3. **a.** Illustration of the procedure for q-range binning for azimuthal integration based on radial integration of the SAXS scattering intensity data. **b.** Influence of CNC concentration on the characteristic volume-averaged spacing at nanoscale at the beginning of shearing, $\dot{\gamma}_{min}$. **c.** Same as for **b.** but for $\dot{\gamma}_{max}$. Note that the later refers here to the highest shear rate attained in each steady shear experiment.

Sample	q_1	q_2 $[\cdot 10^{-2}$ Å$^{-1}]$	q_3
1.9% [B]-CNC	$[0.83, 1.11]$	$[1.15, 1.79]$	$[1.83, 2.99]$
2.5% [B]-CNC	$[0.75, 0.87]$	$[0.91, 1.63]$	$[1.67, 2.99]$
3.9% [B]-CNC	$[1.07, 1.59]$	$[1.63, 2.35]$	$[2.39, 2.99]$
4.4% [B]-CNC	$[1.31, 1.99]$	$[2.03, 2.63]$	$[2.67, 2.99]$
1.2% [P]-CNC	$[0.75, 0.87]$	$[0.91, 1.63]$	$[1.67, 2.99]$
2.5% [P]-CNC	$[1.03, 1.39]$	$[1.43, 2.03]$	$[2.07, 2.99]$
2.5% [P]-CNC-OH-2-Prop-C_2-N-C_2	$[1.03, 1.39]$	$[1.43, 2.03]$	$[2.07, 2.99]$

Table S1. List of azimuthal integration q-ranges. Intervals q_2 have been used in the analysis unless otherwise stated.

SI 5 Additional data

This section includes still frame extracts from PLI video recordings, PLI (L_2, t) space-time diagrams for the data in Fig. 3 and 4, POM and TEM analysis, additional multiscale analysis diagrams, some mentioned in the main manuscript, data comparing order parameters and viscosity functions between different measuring geometries and azimuthal integration q-ranges, see Table S1, and a graphical illustration of the presumed multiscale orientation in biphasic suspension. Figures that contain relevant information supporting the manuscript are further discussed.

SI 5.1 Complementary biphasic B-CNC tests

The multiscale orientation behavior described in Section 2.2 appears to have a lower CNC concentration limit which could be still within the biphasic region. This is apparent already for 1.9% [B]-CNC which in some iterations of our tests had shown non-simultaneous nano-meso orientation depending on the batch. For the case in Fig. S13.b, it would appear that $\dot{\gamma}^{\mathrm{SAXS}}_{\mathrm{orien}} < \dot{\gamma}^{\mathrm{PLI}}_{\mathrm{orien}}$. However, this can be traced to a progressive growth of a Maltese-cross pattern from the outer edge of the geometry, see $\dot{\gamma}^{\mathrm{PLI}}_{r=R}$. Interestingly, when comparing the SAXS order parameter between the q-ranges investigated, it seems that the critical shear rate for nanoscale orientation is higher for both q_1 and q_3. A similar observation could potentially be made also for q_1 for the otherwise simultaneously-aligning across lengthscales 2.5% [P]-CNC. This, together with the very high probe sonication dose and the fact that the two critical shear rates, SAXS and PLI, are equal within q_2 but not as expected based on considering the detection of earlier nanoscale orientation at $\dot{\gamma}(r) > \dot{\gamma}$ could suggest that mesoscale orientation could still drive the structuring of the sample ahead of nanoscale orientation.

Testing an isotropic CNC suspension (based on the POM in Fig. S8), 1.2% [P]-CNC, Fig. S13 a., no multiscale orientation could be detected. However, we note that for integration q-ranges below and above structural peak, weak orientation at nanoscale could be detected, Fig. S12.

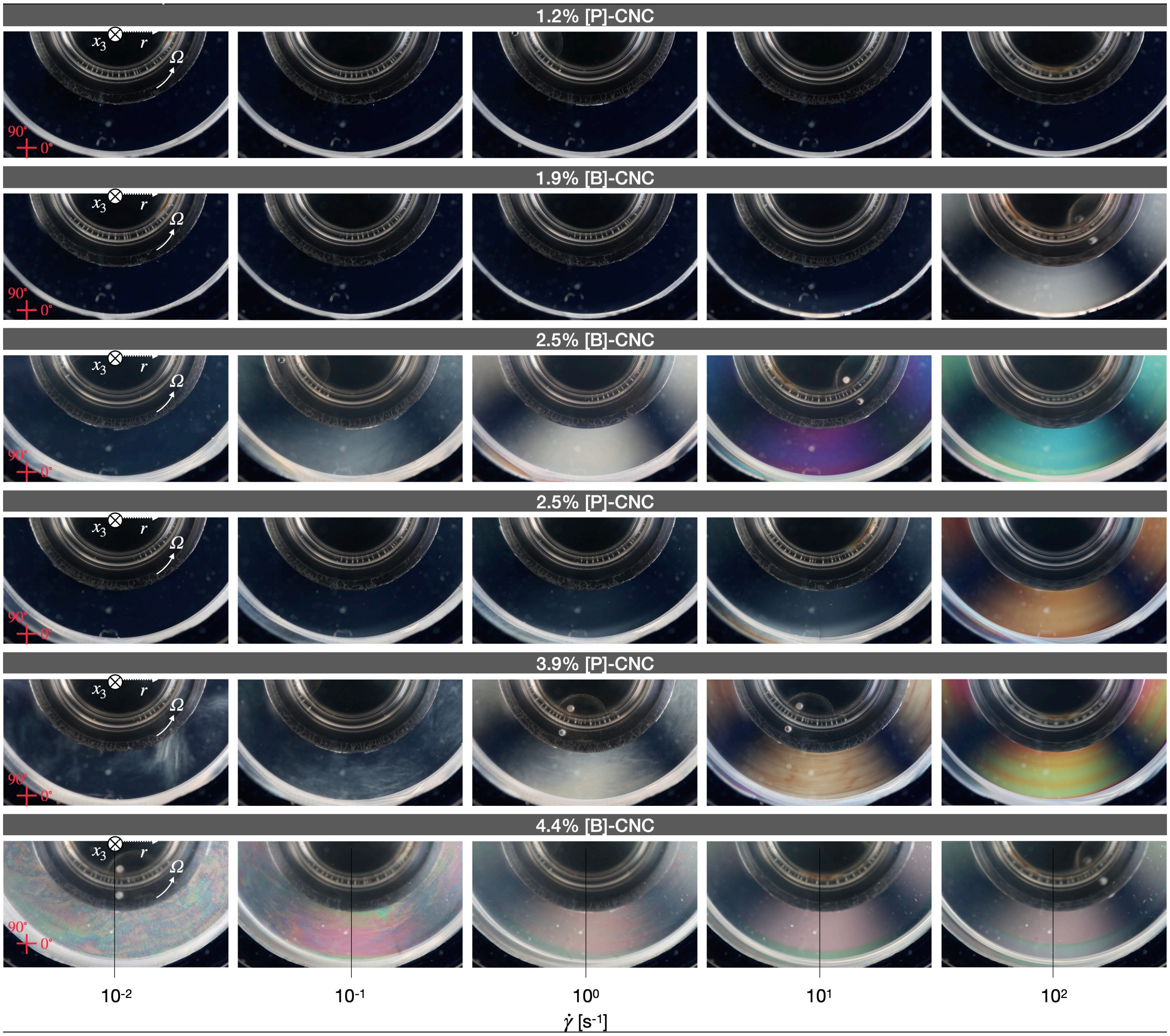

Figure S4. Still frame extracts from PLI video recordings at defined shear rates for all [B] and [P]-CNC suspensions.

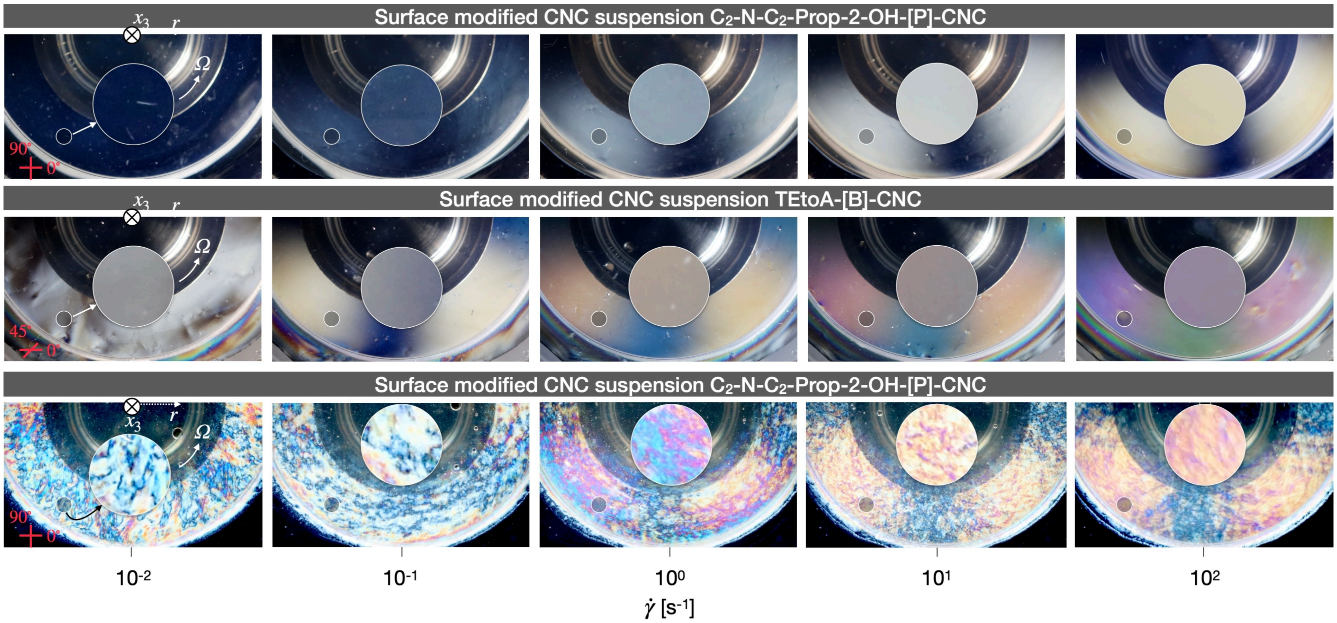

Figure S5. Still frame extracts from PLI video recordings at defined shear rates for the modified CNC suspensions and (L,d)-2NapFF

.

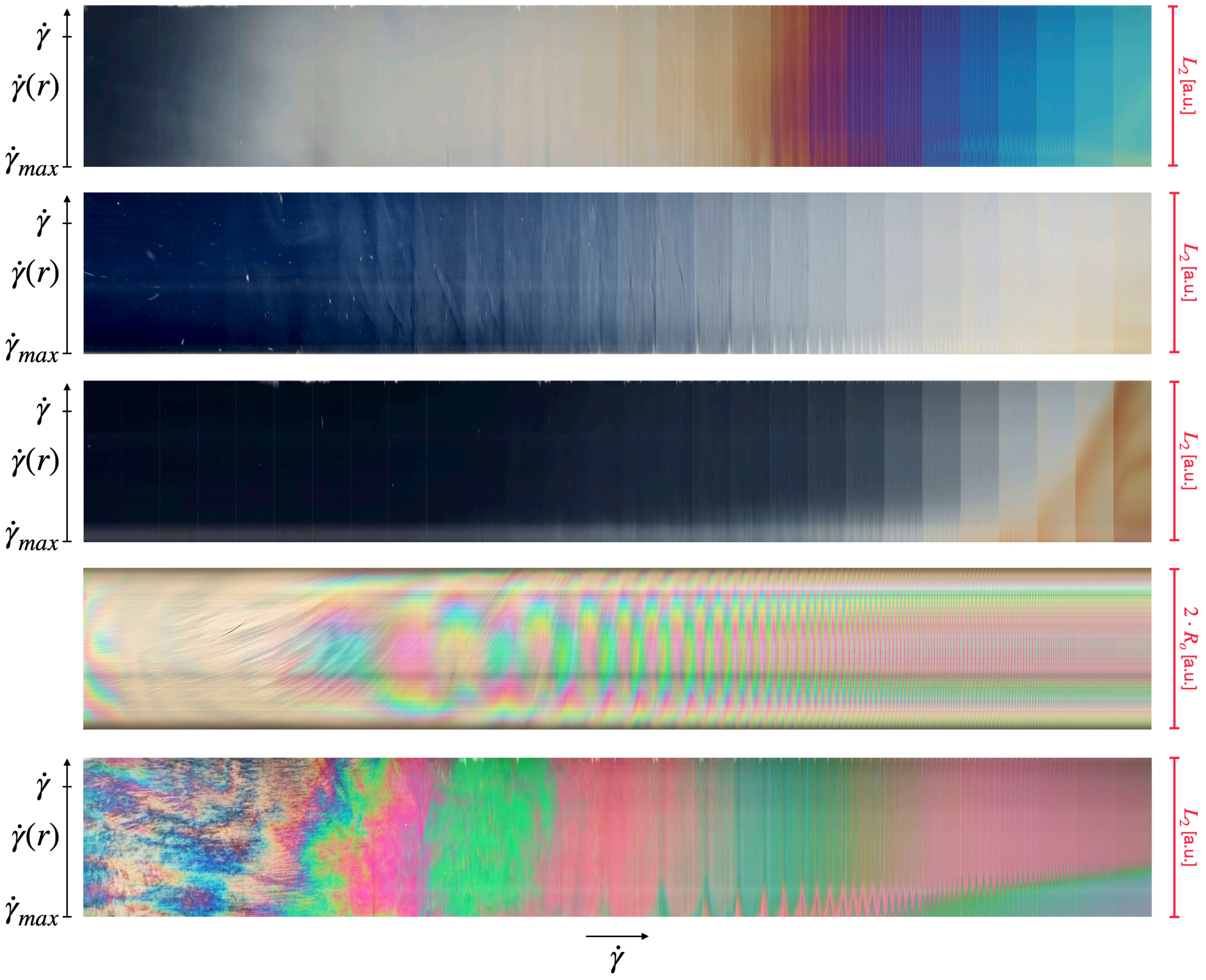

Figure S6. PLI space time diagrams in the orthogonal direction with respect to the data in Fig. 3 and 4. For PP geometries this corresponds to the radial distance L_2 is illustrated in Fig. 2.d.

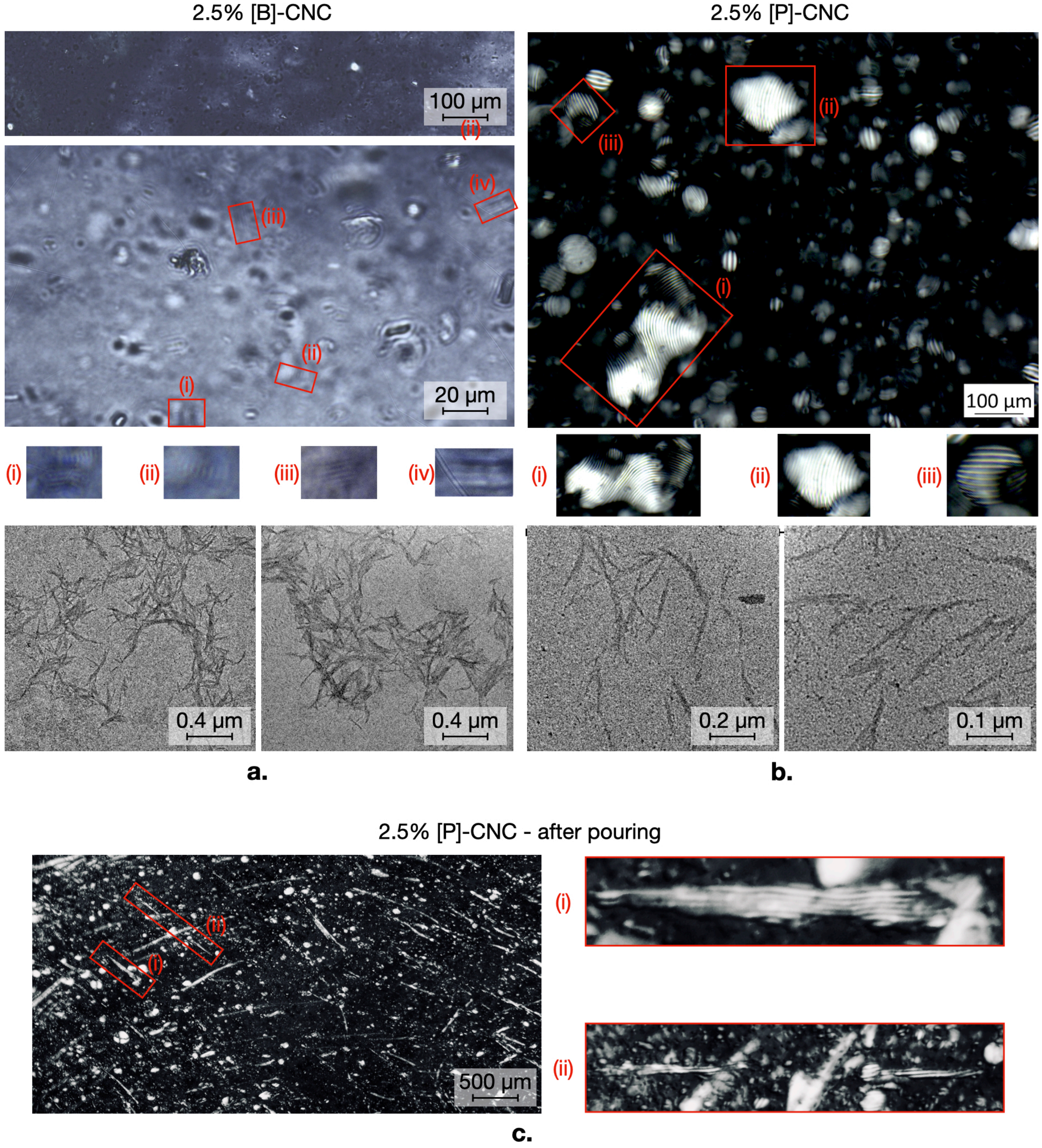

Figure S7. POM and TEM analysis of **a.** 2.5% [B]-CNC and **b.** 2.5% [P]-CNC, and c. 2.5% [P]-CNC showing elongated chiral nematic domains if slightly perturbed by flow.

1.2% [B]-CNC

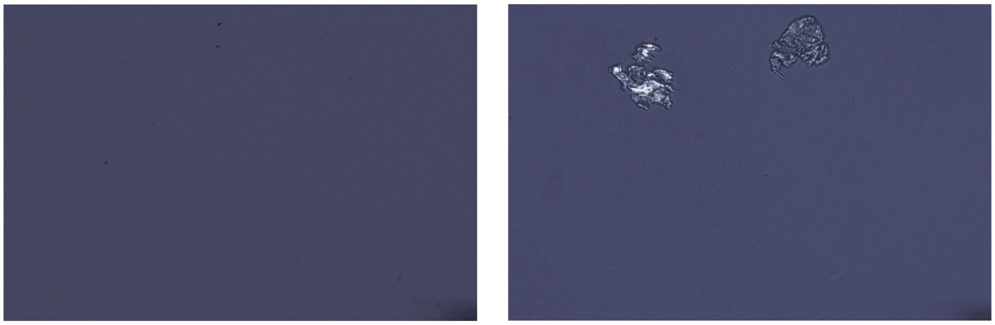

a. b.

Figure S8. POM analysis of 1.2% [B]-CNC.

4.4% [B]-CNC

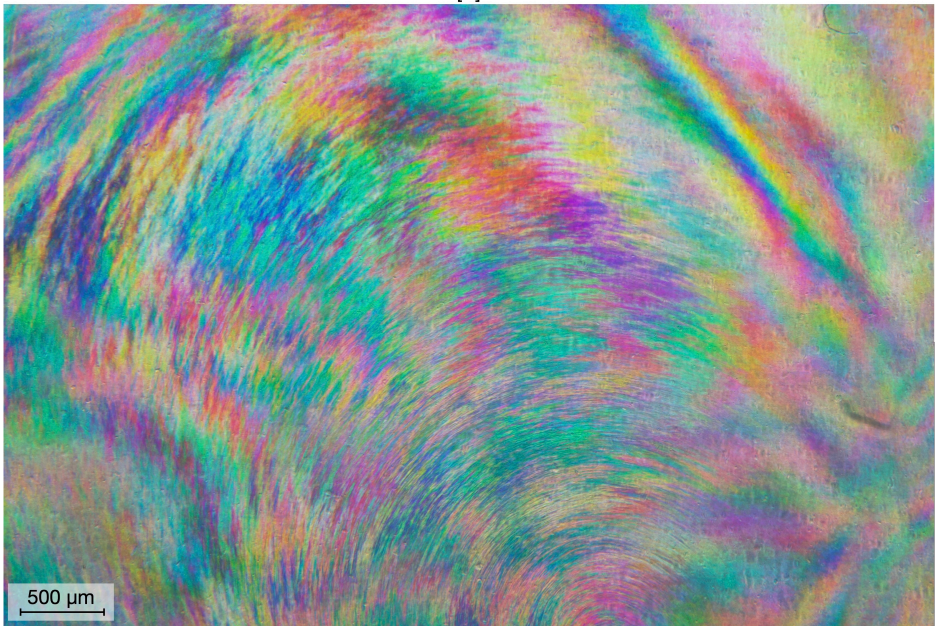

Figure S9. POM of 4.4% [B]-CNC.

RTWN

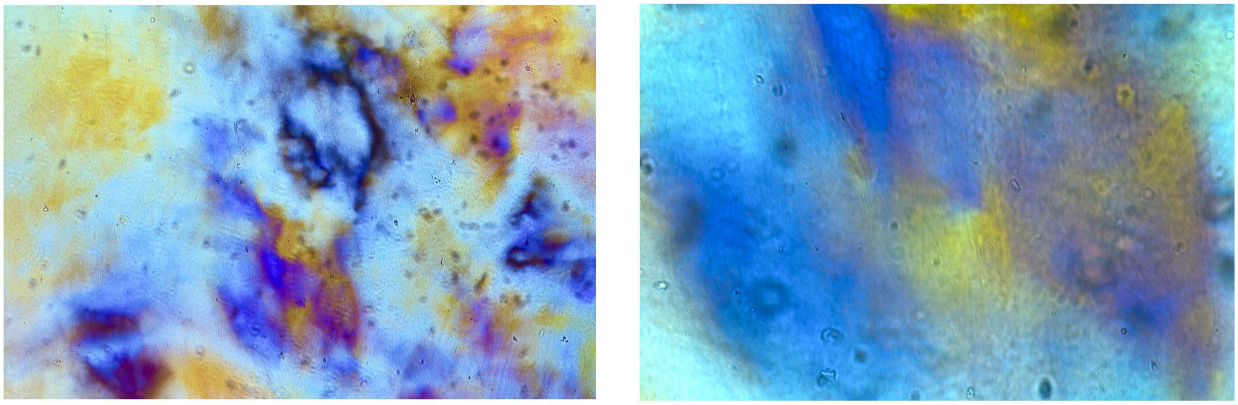

Figure S10. POM of (L,d)-2NapFF
. Cryo-EM of similar compositions can be found in McAulay et al.[35].

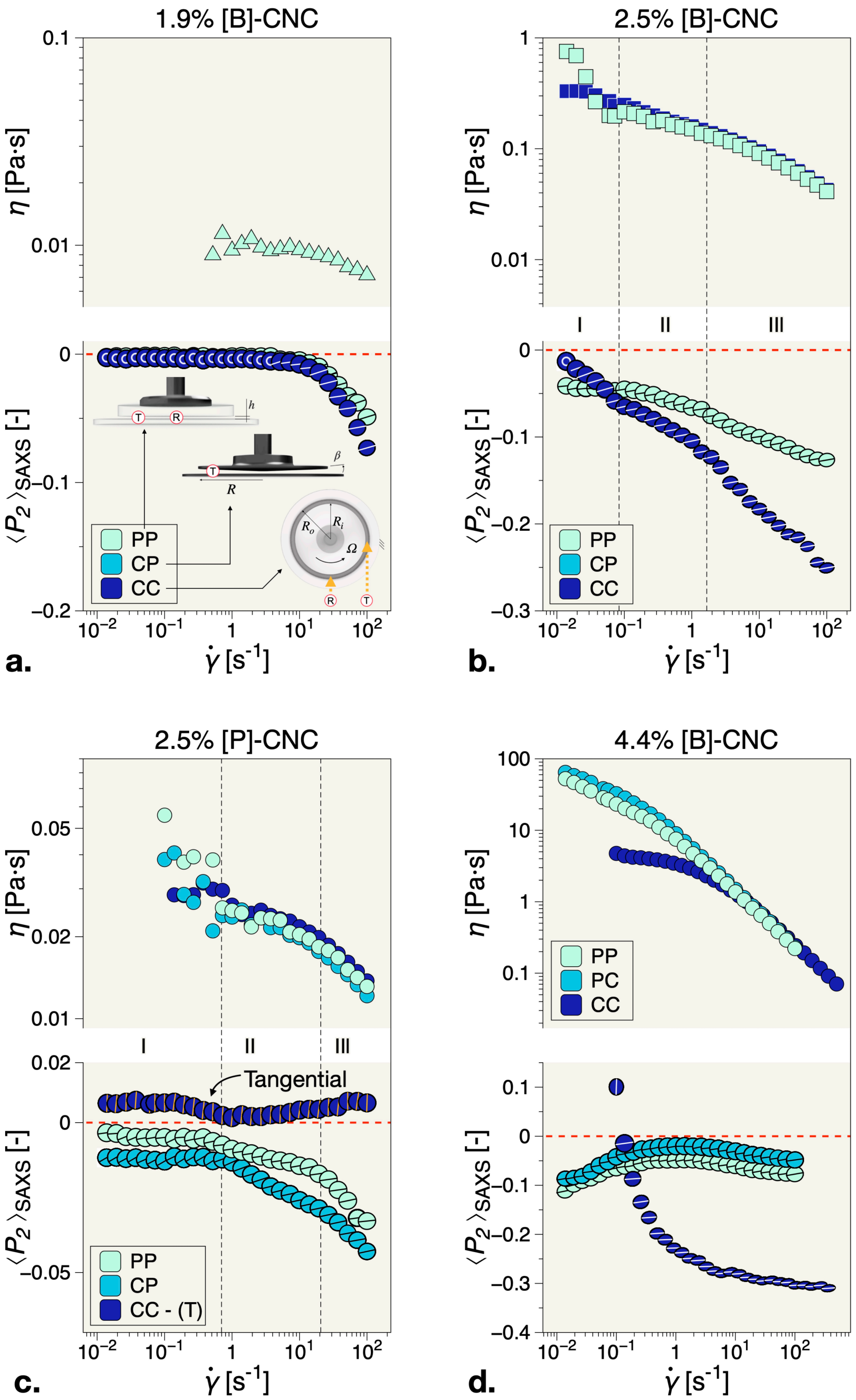

Figure S11. Comparison between SAXS order parameters and viscosity functions from Rheo-SAXS measurements performed on: PP - parallel plate geometry, where a radial incident beam in the (1)-(2) plane (velocity - velocity gradient) crosses a shear rate gradient from a maximum at the outer edge of the geometry to zero in the center, CC - concentric cylinder measuring geometry (the standard in the field) where a radial incident beam in the (1)-(3) plane (velocity - vorticity) the thin gap between the concentric of constant shear rate and CP - cone - plate measuring geometry, where the shear rate is constant in the radial direction, however a tangential incident X-ray beam crosses a range of nanoparticle orientations for the same shear rate.

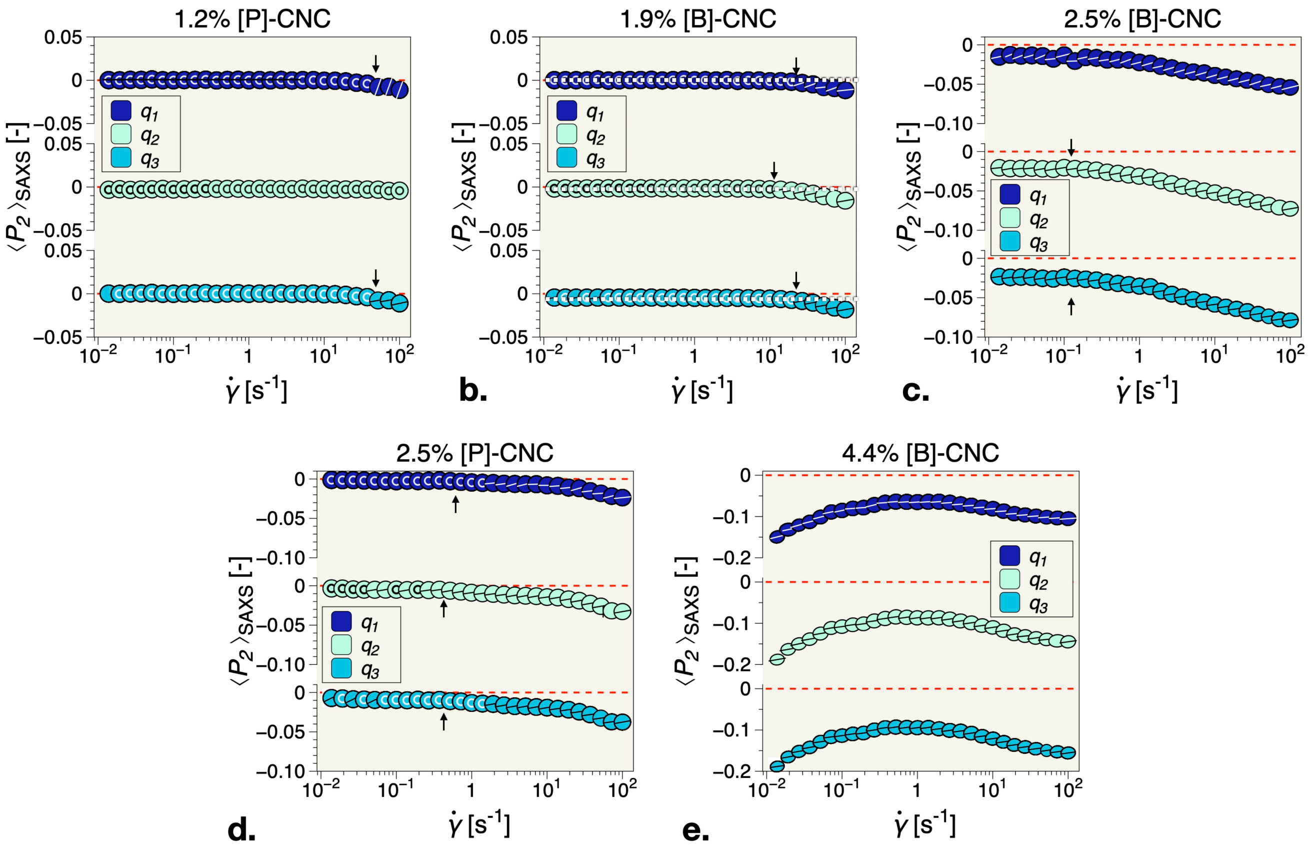

Figure S12. Comparison between Hermans order parameters obtained using q_1, q_2 and q_3 binning ranges, see Table S1. Note that the threshold for isotropic limit, i.e. the value of $\langle P_2 \rangle_{SAXS}$ below which based on inspecting the respective azimuthal integrations, is not consistent within this figure.

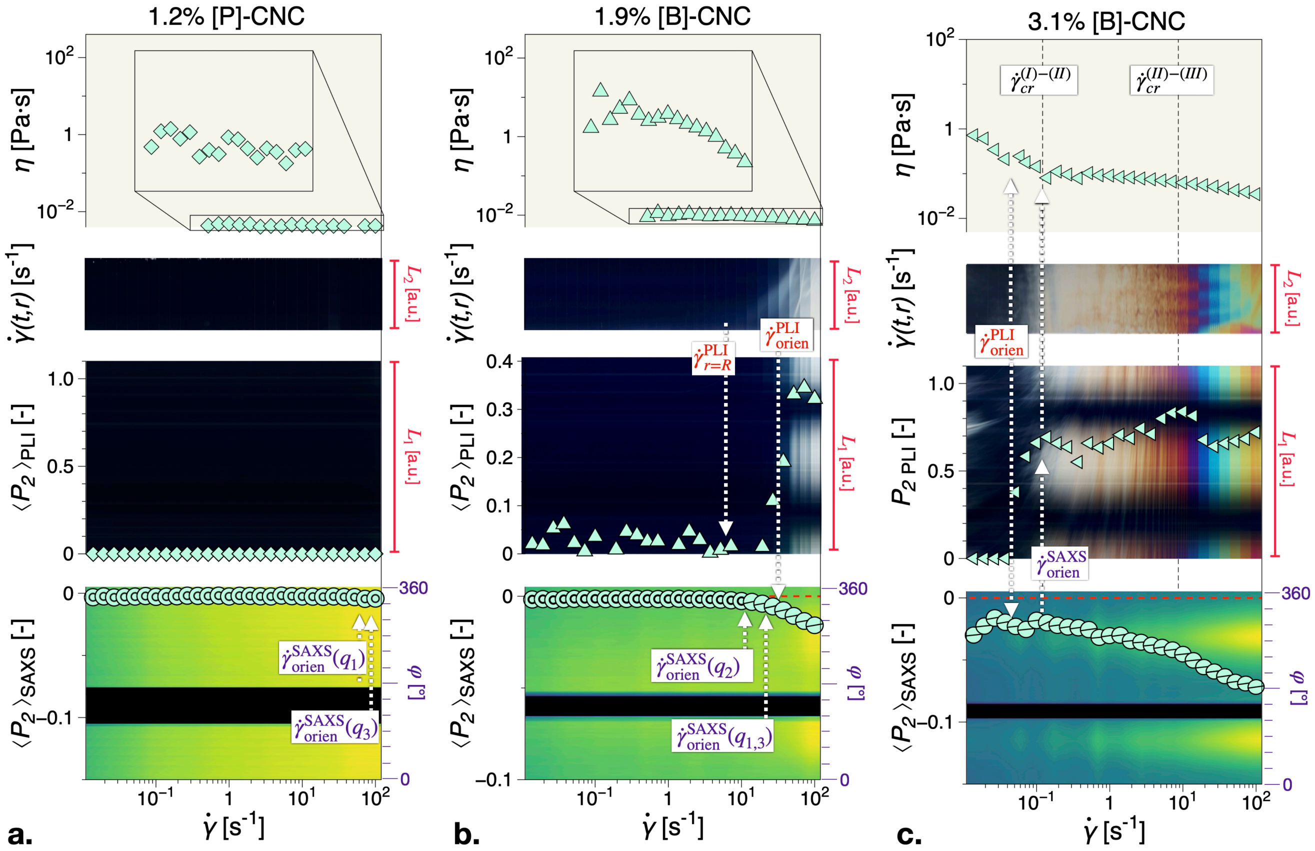

Figure S13. Some of the additional CNC suspensions investigated: **a.** 1.2% [P]-CNC, **b.** 1.9% [B]-CNC and **c.** 3.9% [B]-CNC. Notably, the biphasic 1.9% [B]-CNC in **b**. is somewhat similar in viscosity with 2.5% [P]-CNC, however, if we consider space-time diagrams in L_2, see Fig. 2.d, it is clear that a Maltese-cross pattern is still visible before orientation in the flow direction at nanoscale ensues. We note here also that in this case the critical shear rate for nanoscale orientation depends on the integrated q-range.

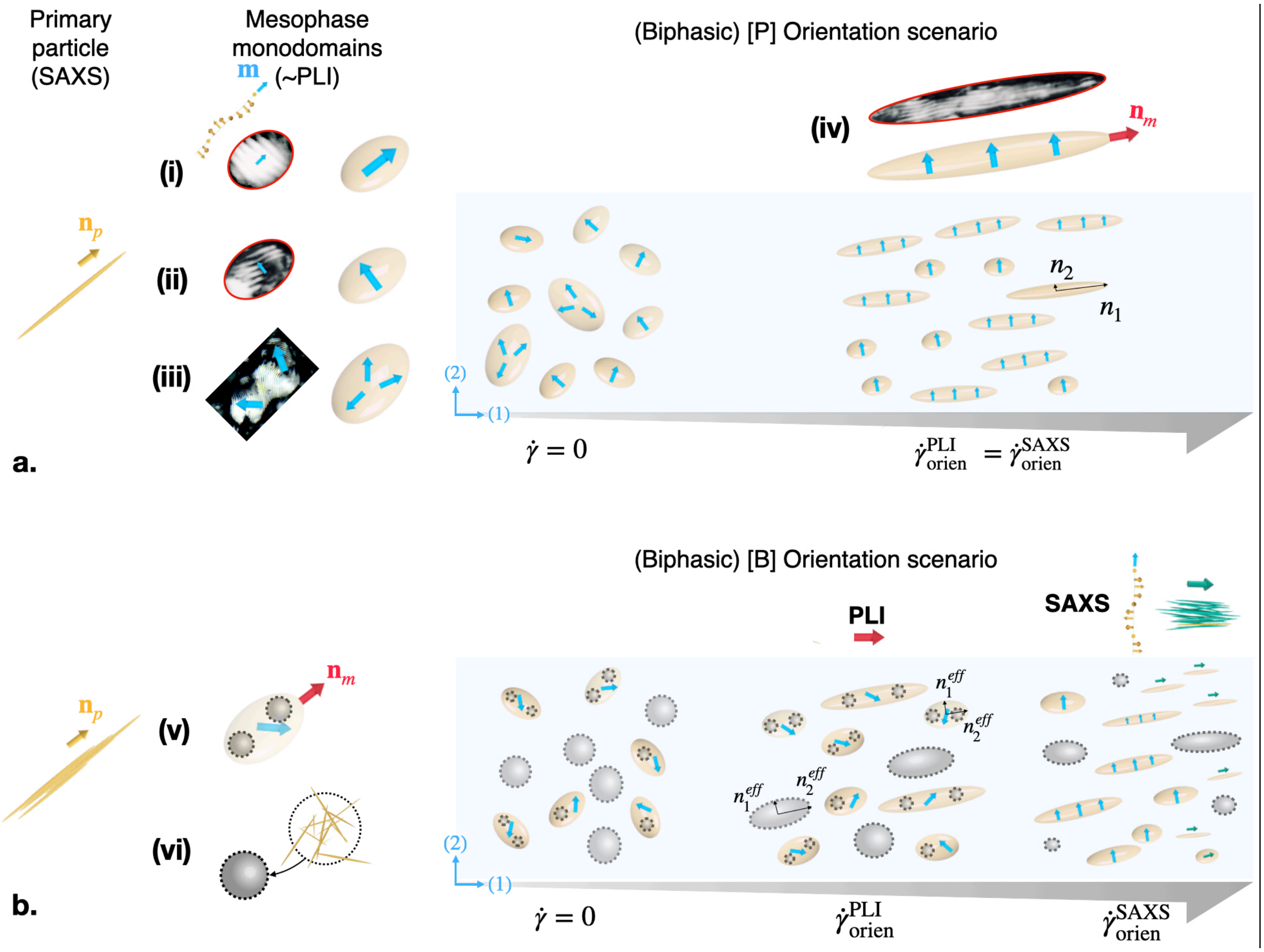

Figure S14. Illustration of the multiscale flow-induced structuring of biphasic CNC suspensions: **a**. for CNC suspensions where composed predominantly of primary CNC crystallites and their higher order assemblies **b**. for CNC suspensions composed predominantly of CNC bundles and aggregates and their higher order assemblies and agglomerates.

SI 5.2 Comparing stripes in rheo-PLI-SAXS parallel-plate and concentric-cylinder measuring geometries

To understand the formation of stripes in 4.4% [B]-CNC and how it relates to nanostructure alignment we compare the rheo-PLI-SAXS experiments in parallel-plate measuring setup with a custom concentric cylinder (CC) system, Fig. 5. A still-frame visualization of the CC Rheo-PLI-SAXS setup is presented in Fig. S15.

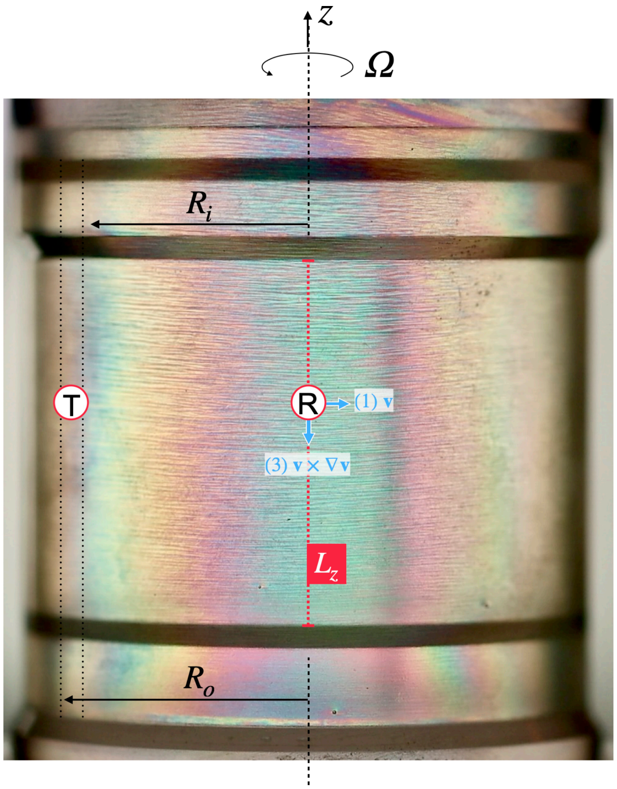

Figure S15. Annotated visualization of the concentric cylinders Rheo-PLI-SAXS geometry as seen through the polarized light optical train. Line L_z is used in the Fig. 4.b.

Similar types of PLI textures in the form of stripes are observed in both cases with orientations according to the flow induced by the gap setting / measurement position setting. While $\langle P_2 \rangle_{\mathrm{SAXS}}$ in PP attains a relatively high value at the beginning of shear, $\langle P_2 \rangle_{\mathrm{SAXS}} < 0$, in CC it is above zero, i.e., $\langle P_2 \rangle_{\mathrm{SAXS}} > 0$ but with a considerably lower value. This would be consistent with previous depictions of roll formation in the director flow patterns[39], with the note that in PP x-rays probe in the plane of the stripes while in CC x-rays probe perpendicular to their plane. Within the transient motion to steady state in the first two datapoints measured, the stripes appear to roll in the azimuthal direction at first, whereafter the director field reforms into stripes in the shearing direction at $\dot{\gamma}_{(3)\rightarrow(1)}$, reaching a steady structure at $\dot{\gamma}_{(1)}$. Both critical shear rates mentioned correspond to changes in $\langle P_2 \rangle$ de-orientation rates (slopes in the decrease in order parameter). Importantly, the onset of the Maltese-cross pattern, $\dot{\gamma}^{\mathrm{PLI}}_{\mathrm{orien}}$, is detected during de-orientation at nanoscale and approximately one decade in shear rate before an increase in preferential orientation in the flow direction at nanoscale can be detected. Based on the de-structuring rates (slopes in $\langle P_2 \rangle_{SAXS}$), two regimes can be distinguished. They can be broadly associated with initial structural configuration for $\dot{\gamma} < \dot{\gamma}^{\mathrm{PLI}}_{\mathrm{orien}}$ to decrease in the de-structuring rate at $\dot{\gamma}^{\mathrm{PLI}}_{\mathrm{orien}}$ until nanoscale orientation ensues at $\dot{\gamma}^{SAXS}_{\mathrm{orien}}$. This further underlines that in the PP Rheo-PLI-SAXS experiment a clear sequence of nano-meso/macro physical processes can be evidenced. By comparison, in CC the increase in shear rate is rather uneventful with just a monotonic decrease in the SAXS order parameter.

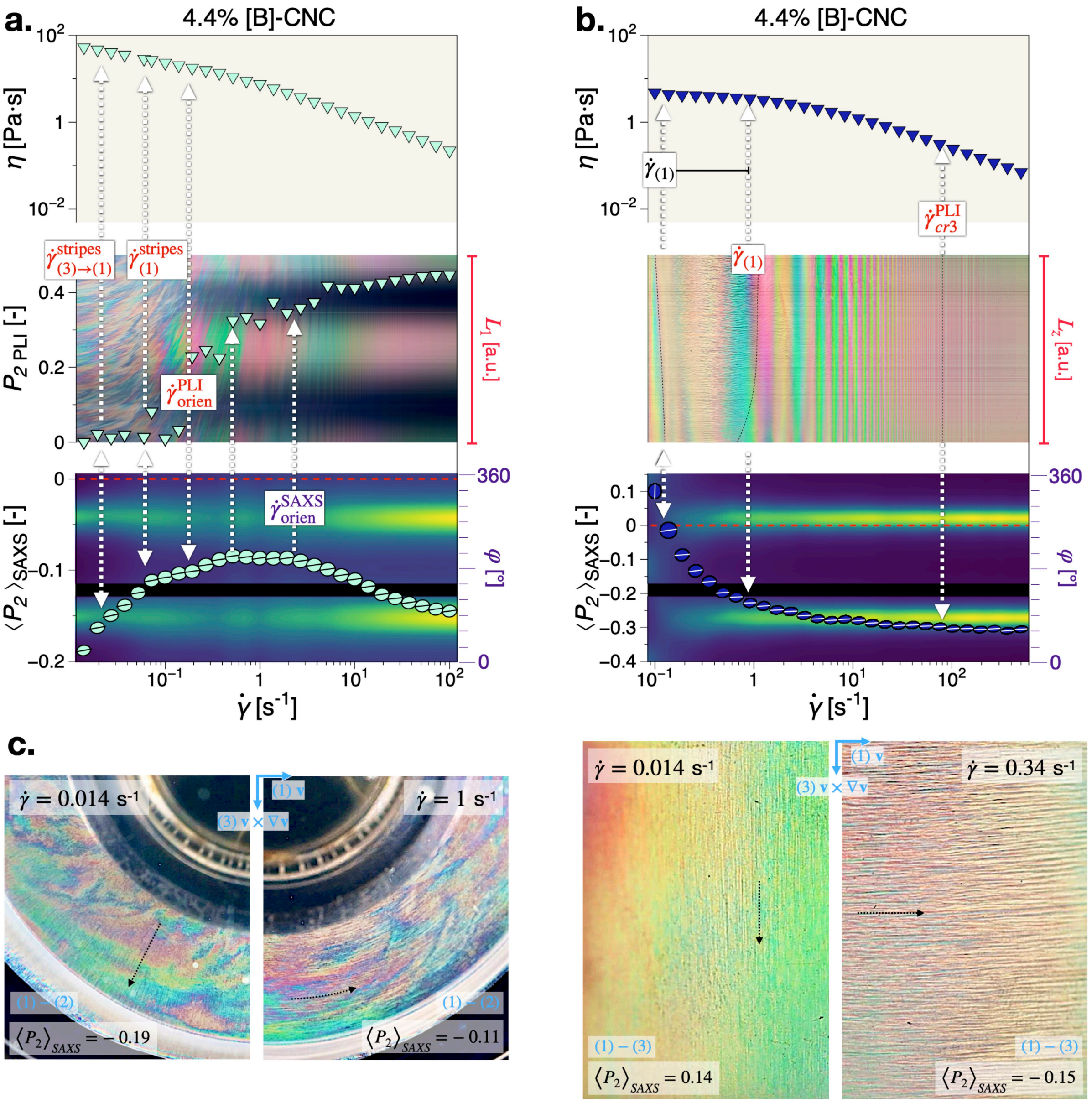

Figure S16. **a**. Multiscale analysis (see Fig. 2.a) of 4.4% [B]-CNC (parallel-plate) an d **b**. the same concentration observed in a Rheo-PLI-SAXS concentric cylinders experiment; **c**. comparison of PLI textures observed in parallel-plate tests and concentric-cylinder tests.
